# Mechanisms of Qingyi Decoction in Severe Acute Pancreatitis-Associated Acute Lung Injury via Gut Microbiota: Targeting the Short-Chain Fatty Acids-Mediated AMPK/NF-κB/NLRP3 Pathway

**DOI:** 10.1128/spectrum.03664-22

**Published:** 2023-06-20

**Authors:** Zhengjian Wang, Jin Liu, Fan Li, Shurong Ma, Liang Zhao, Peng Ge, Haiyun Wen, Yibo Zhang, Xiaojun Liu, Yalan Luo, Jiaqi Yao, Guixin Zhang, Hailong Chen

**Affiliations:** a Department of General Surgery, The First Affiliated Hospital of Dalian Medical University, Dalian, Liaoning, People’s Republic of China; b Institute (College) of Integrative Medicine, Dalian Medical University, Dalian, Liaoning, People’s Republic of China; c Laboratory of Integrative Medicine, The First Affiliated Hospital of Dalian Medical University, Dalian, Liaoning, People’s Republic of China; d Department of Anesthesiology, First Affiliated Hospital of Dalian Medical University, Dalian, Liaoning, People’s Republic of China; 南昌大学

**Keywords:** AMPK/NF-κB/NLRP3 pathway., gut microbiota, gut-lung axis, Qingyi decoction, severe acute pancreatitis-associated acute lung injury, short-chain fatty acids

## Abstract

The pivotal roles of gut microbiota in severe acute pancreatitis-associated acute lung injury (SAP-ALI) are increasingly revealed, and recent discoveries in the gut-lung axis have provided potential approaches for treating SAP-ALI. Qingyi decoction (QYD), a traditional Chinese medicine (TCM), is commonly used in clinical to treat SAP-ALI. However, the underlying mechanisms remain to be fully elucidated. Herein, by using a caerulein plus lipopolysaccharide (LPS)-induced SAP-ALI mice model and antibiotics (Abx) cocktail-induced pseudogermfree mice model, we tried to uncover the roles of the gut microbiota by administration of QYD and explored its possible mechanisms. Immunohistochemical results showed that the severity of SAP-ALI and intestinal barrier functions could be affected by the relative depletion of intestinal bacteria. The composition of gut microbiota was partially recovered after QYD treatment with decreased *Firmicutes*/*Bacteroidetes* ratio and increased relative abundance in short-chain fatty acids (SCFAs)-producing bacteria. Correspondingly increased levels of SCFAs (especially propionate and butyrate) in feces, gut, serum, and lungs were observed, generally consistent with changes in microbes. Western-blot analysis and RT-qPCR results indicated that the AMPK/NF-κB/NLRP3 signaling pathway was activated after oral administration of QYD, which was found to be possibly related to the regulatory effects on SCFAs in the intestine and lungs. In conclusion, our study provides new insights into treating SAP-ALI through modulating the gut microbiota and has prospective practical value for clinical use in the future.

**IMPORTANCE** Gut microbiota affects the severity of SAP-ALI and intestinal barrier function. During SAP, a significant increase in the relative abundance of gut pathogens (Escherichia, *Enterococcus*, Enterobacter, *Peptostreptococcus*, *Helicobacter*) was observed. At the same time, QYD treatment decreased pathogenic bacteria and increased the relative abundance of SCFAs-producing bacteria (*Bacteroides*, *Roseburia*, *Parabacteroides*, *Prevotella*, *Akkermansia*). In addition, The AMPK/NF-κB/NLRP3 pathway mediated by SCFAs along the gut-lung axis may play an essential role in preventing the pathogenesis of SAP-ALI, which allows for reduced systemic inflammation and restoration of the intestinal barrier.

## INTRODUCTION

Severe acute pancreatitis (SAP) is one of the most common emergencies in general surgery, mainly caused by biliary stones, overeating, excessive alcohol consumption, hyperlipidemia, and abdominal infection (1). SAP is frequently accompanied by systemic inflammatory response syndrome, endotoxemia, and multiple organ failure, with an overall 20 to 40% fatality rate (2). Acute lung injury (ALI) is the first complication secondary to SAP, with an incidence rate of 27.7% and a 60% fatality rate (3). A cascade of mechanisms, including pancreatic necrosis, immune imbalance, loss of intestinal barrier integrity, and sepsis, have been reported to be involved in the pathogenesis of severe acute pancreatitis-associated acute lung injury (SAP-ALI); however, the exact etiology has not been fully elucidated (4–8).

In recent years, studies have suggested that gut microbiota plays a crucial role in the pathogenesis of SAP-ALI (9). An imbalanced ratio of beneficial/pathogenic bacteria in the gut occurs in the progression of SAP (10). Upon destroying the intestinal barrier, intestinal bacteria especially those with pathogen-associated molecular patterns (PAMPs) can be translocated through the mucosal lamina propria to the lungs, thus triggering a sustained proinflammatory immune response resulting in the release of inflammatory factors, such as interleukin-1β (IL-1β), interleukin-6 (IL-6), tumor necrosis factor-α (TNF-α) (11). Therefore, the bidirectional cross talk between the gut microbiota and lungs called the gut-lung axis, has been considered a target for treating SAP-ALI (12–14).

Short-chain fatty acids (SCFAs), specifically acetate, propionate, and butyrate, have recently been discovered to play roles as vital mediators in the gut-lung axis, and several studies have shown that the exhaustion or death of SCFA-producing bacteria in the gut has the potential to accelerate the progression of SAP (14, 15). A recent study comparing levels of SCFAs in the cecal feces of SAP mice and wild mice showed that the concentration of SCFAs, specifically butyrate, was significantly reduced in the SAP group (16). A decrease in the initial content and production rate of SCFAs was also observed in the colon of germfree mice, suggesting the possibility of delaying the pathogenesis of SAP-ALI via supplementation with dietary fiber and specific forms of bacteria (17). Furthermore, SCFAs can serve as the substrates for energy metabolism in the tricarboxylic acid cycle and regulate AMP (AMP)-activated protein kinase (AMPK) to maintain internal environmental homeostasis (18). Activation of AMPK is mediated via phosphorylation of the T172 site on the alpha subunit, and this phosphorylated AMPK (p-AMPK) can regulate the phosphorylation of downstream target proteins, such as nuclear factor-kappa B (NF-κB) and thus participates in a variety of host life processes, including energy metabolism, immunity, inflammatory response, and autophagy (19). The AMPK/NF-κB pathway plays a crucial role in repairing mitochondrial function and promoting the renewal and repair of epithelial cells, which helps maintain the integrity of the intestinal barrier and the air-blood barrier (20, 21). Additionally, the AMPK/NF-κB/NOD-like receptor family pyrin domain containing 3 (NLRP3) pathway has been found to attenuate systemic inflammation and oxidative stress, the mechanism of which mainly involves inhibition of proinflammatory cytokines that are released by macrophages (22). Therefore, maintaining intestinal bacterial (and immune) homeostasis and subsequent recovery from a proinflammatory state are thus considered prospective strategies to protect against SAP-ALI.

Qingyi decoction (QYD), a traditional Chinese medicine (TCM) commonly used for treating SAP in the clinic, consists of eight herbs, including *Rhubarb*, *Radix Bupleuri*, *Radix Aucklandiae*, *Paeoniae Radix Alba*, *Natrii Sulfas*, *Rhizome Corydalis*, *Gardenia jasminoides*, and *Scutellaria baicalensis Georgi* (23). QYD has many pharmacological activities, including a role as an antiinflammatory, antioxidant, immune regulatory effector, a player in intestinal barrier recovery, and a promoter of cell apoptosis (24–26). It has been shown that QYD alleviates the systemic inflammatory responses by inhibiting the activation of NLRP3 inflammasomes in macrophages and regulating the expression of secreted phospholipase A2 and G-protein-coupled bile acid receptors in SAP (25, 27). Furthermore, our previous study suggested that QYD could potentially reduce damage-associated molecular patterns during SAP by inhibiting high mobility group box-1 (28). Despite the multicomponent, multitarget, and multipathway characteristics of QYD, its mechanism for the treatment of SAP-ALI still needs to be better understood.

In this study, we aim to investigate the mechanisms and roles of the gut microbiota and its derived metabolites (SCFAs) for QYD treatment of SAP-ALI. The severity of SAP-ALI and the intestinal barrier's functions were influenced by gut microbiota alterations, resulting in pathological changes and damage to the pancreas, intestine, lungs, and systemic inflammatory response. Compared to the SAP group, we found that QYD treatment enhanced intestinal barrier function, alleviated symptoms of SAP-ALI, and increased the relative abundance of SCFAs-producing bacteria. These results were consistent with the trend of targeted metabolomics for detecting SCFAs in multiple tissues associated with the gut-lung axis, particularly propionate and butyrate. The regulations on the AMPK/NF-κB/NLRP3 signaling pathway after treatment with QYD were further confirmed by Western-blot, qPCR analysis, and immunofluorescence, exhibiting close correlations with the effects on SCFAs in the intestine and lungs. Thus, these data indicated that the mechanisms of QYD for treating SAP-ALI via altering the gut microbiota may be associated with SCFAs-mediated AMPK/NF-κB/NLRP3 signaling pathway.

## RESULTS

### Deficiency of gut microbiota affects the severity of SAP-ALI, whereas QYD treatment alleviates the symptoms of SAP-ALI in SAP mice.

**FIG 1:**
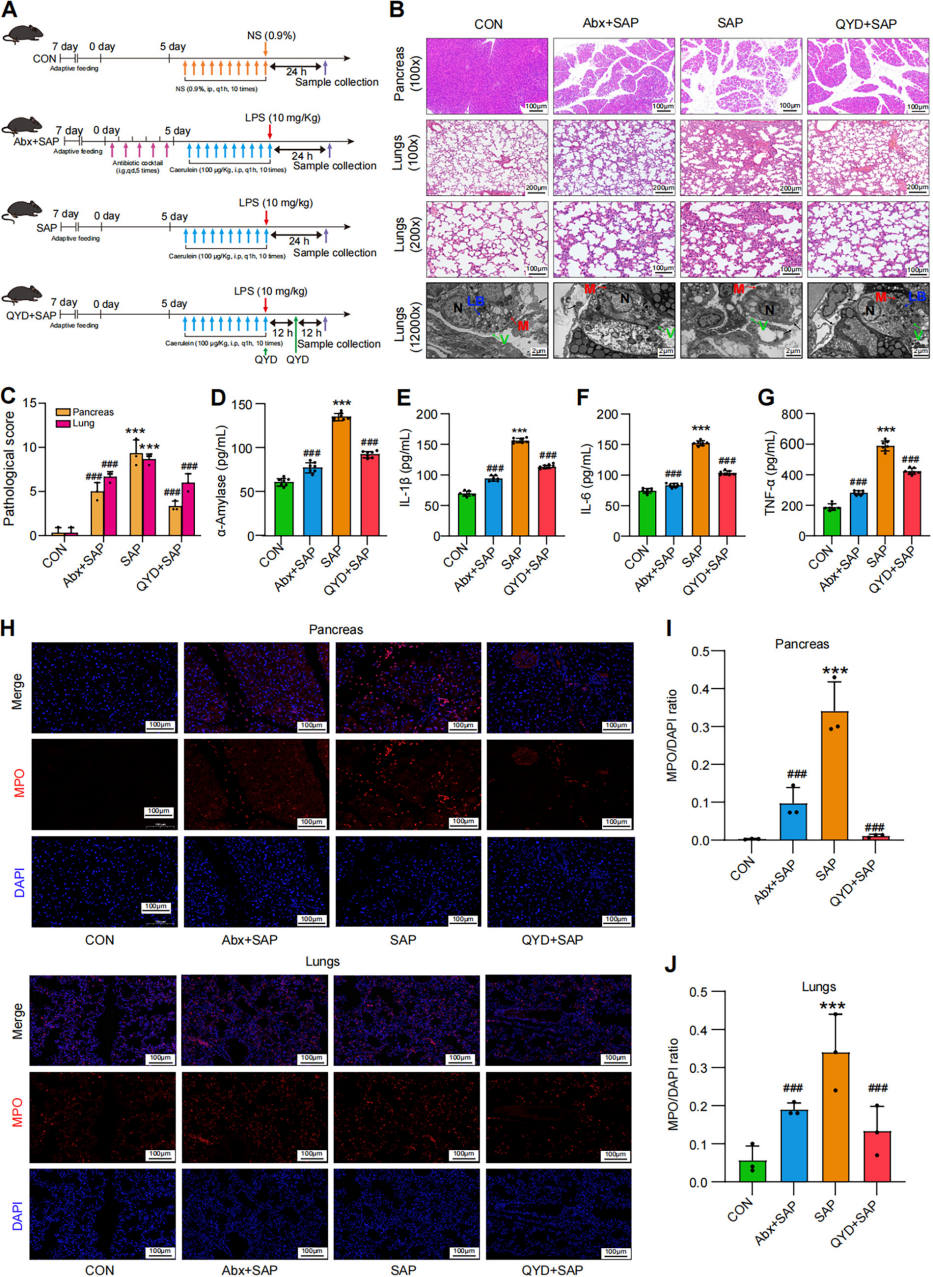
Relative depletion of intestinal bacteria influenced the severity of SAP-ALI, while QYD treatment alleviated the pathological damage to pancreatic and lung tissue as well as the systemic inflammatory response in the caerulein combined with the LPS-induced SAP-ALI model in mice. (A) Overall experimental design and grouping. (B) Representative H&E staining of pancreas and lungs. Samples used microscopy (100× and 200× magnification) and transmission electron microscopy (original magnification, 12,000×). LB: lamellar bodies, M: mitochondria, N: nuclear, V: villus; black arrow: air-blood barrier. (C) Pathological damage score of pancreas and lungs. (D to G) Serum expressional levels of α-amylase, IL-1β, IL-6, TNF-α by ELISA. (H) Immunofluorescence staining results for MPO (red), nucleus (DAPI, blue), and merged images in pancreas and lungs. Scale bar: 100 μm. (I to J) Semiquantitative results of MPO in pancreas and lungs. Data are representative images or shown as mean ± SEM for each group of mice (*n* = 7 per group) with at least three independent experiments and analyzed by unpaired Student's *t* test with ***, *P < *0.001 in comparison with the CON group and **^###^**, *P < *0.001 in comparison with the SAP group.

Caerulein is an analog of cholecystokinin that affects gallbladder contraction and stimulates pancreatic enzyme secretion (29). It is also responsible for activating trypsin from trypsinogen, which can lead to autolysis of pancreatic acinar cells, thus inducing pancreatitis (30). Lipopolysaccharide (LPS) is a type of endotoxin that stimulates the systemic inflammatory response via the activation of monocytes, which in turn release cytokines (31). Therefore, the combined use of caerulein and LPS synergizes in creating a mouse model miming SAP-ALI (32). First, to evaluate the pathology of SAP-ALI, the SAP mice model was established via continuous injections of caerulein and a single dose of LPS (Fig. 1A). Second, prior to constructing the SAP model, mice in the Abx+SAP group were pregavaged with a mixture of four antibiotics (Abx) to relatively deplete the gut bacteria, thus exploring whether gut microbiota would affect the severity of SAP-ALI. The QYD+SAP group aimed to explore whether QYD can alleviate SAP-ALI symptoms and the systemic inflammatory response.

Compared with the control (CON) group, the hematoxylin and eosin (H&E) staining results showed a significant widening of the pancreatic interlobular space, interstitial edema of the pancreatic and lungs with massive inflammatory cell infiltration and punctate hemorrhage, as well as a significantly higher histopathological damage score in the SAP-modeled group (*P < *0.001), confirming the successful induction of SAP-ALI (Fig. 1B and C). Transmission electron microscopy (TEM) showed the partial failure of the air-blood barrier in the lungs of the SAP-ALI group, atrophy of the villi on the surface of type-II alveolar epithelial cells (type-II AECs), structural disorder of lamellar bodies and destruction of mitochondrial cristae (Fig. 1B). Other pathological indicators, specifically serum α-amylase, IL-1β, IL-6, TNF-α, as well as myeloperoxidase (MPO) expression levels in the pancreas and lungs, were significantly upregulated after the construction of SAP, indicating activation of the systemic inflammatory response (Fig. 1D and J). Compared with the SAP group, mice in the Abx+SAP group showed reduced symptoms of SAP-ALI due to pregavage of Abx, including restoration of pulmonary ultrastructure and reduction of α-amylase and inflammation levels, indicating that the gut microbiota plays a vital role in SAP-ALI. Meanwhile, immunofluorescence results showed that the expression levels of MPO associated with neutrophil infiltration in the pancreas and lungs of the Abx+SAP group significantly decreased, which reflects an overall drop in the level of the proinflammatory immune response (Fig. 1H and J). In addition, pathological damage scores and MPO expression in the pancreas and lungs as well as serum inflammatory factor levels were decreased in the QYD-treated group compared with the SAP group (*P < *0.001), suggesting that QYD has the potential to alleviate SAP-ALI symptoms and reduce the systemic inflammatory response (Fig. 1C–J). In more detail, QYD treatment resulted in reduced edema and narrowing of the interlobular space in the pancreas, partial restoration of the air-blood barrier in lung tissue, and protection of mitochondrial integrity (Fig. 1B). The results above proved the progress and severity of SAP-ALI associated with the status of gut microbiota. Additionally, QYD could decrease the inflammatory response in mice, and alleviate damage to the pancreas and lungs, thus mitigating symptoms of SAP-ALI.

### The gut microbiota was altered during SAP-ALI and QYD treatment increased the relative abundance of SCFAs-producing bacteria in mice.

**FIG 2:**
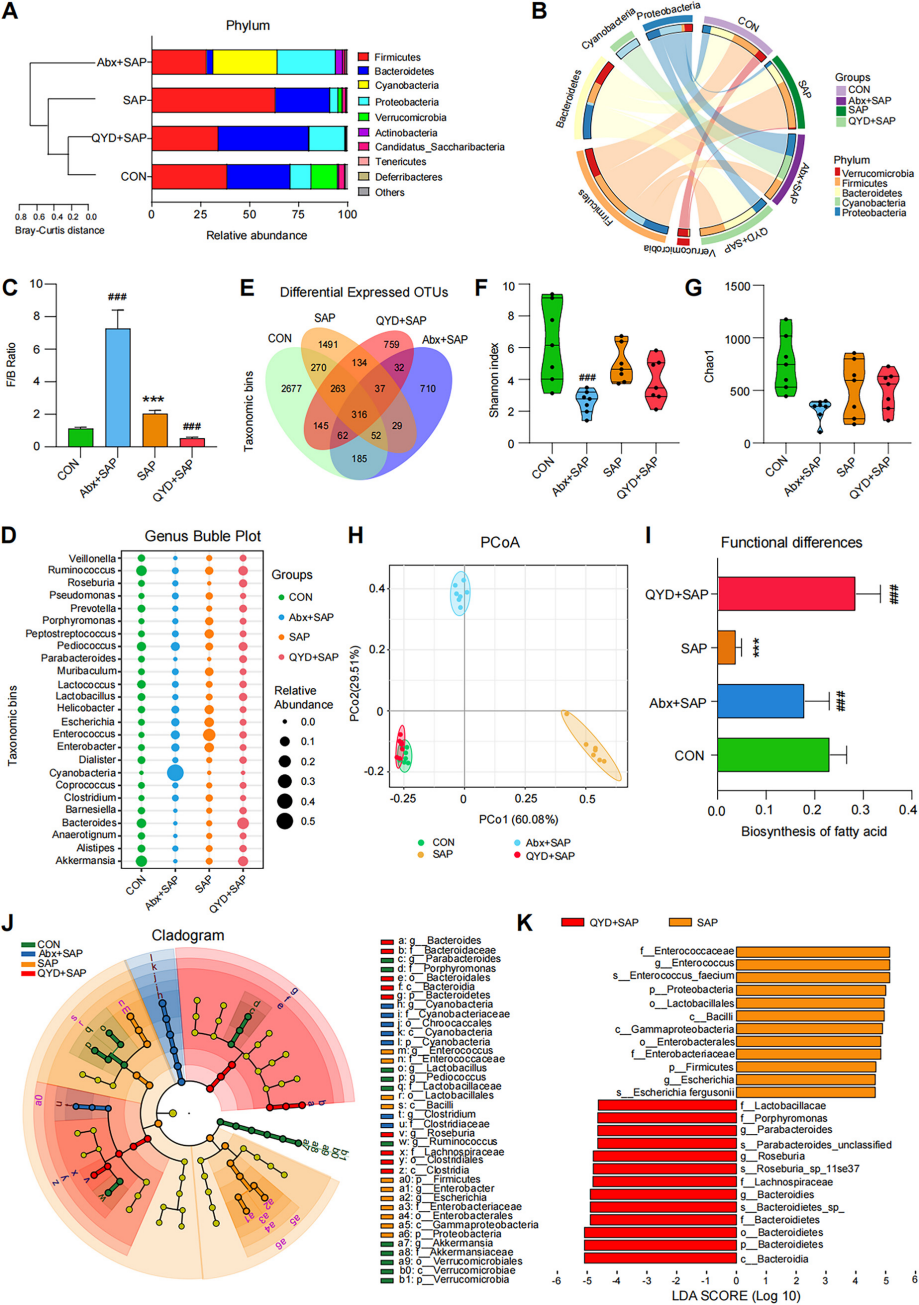
Composition and functional analysis of gut microbiota based on 16S rRNA sequencing. (A) Stacking plot analysis of bacteria at the phylum level using Bray-Curtis distance for clustering aims to show the proportion of the top 10 species in the ranking and the changing trend. (B) Circos diagram of each group at the phylum level (top 5). (C) The ratio of *Firmicutes* to *Bacteroidetes* at the phylum level. (D) Bubble plot of bacteria at the genus level in different groups. (E) Venn diagram based on the abundance of OTUs. The numbers represent the values of OTUs that can be detected in all mice in a group. (F and G) Alpha-diversity analysis of intestinal bacteria at the genus level (Shannon index, Chao1). (H) PCoA plot based on weighted Unifrac distance matrix analysis of the top 25 bacteria. (I) Histogram of differences in fatty acid synthesis based on PICRUSt2 functional predictions. (J-K) Cladogram and distribution histograms showing the results of LEfSe analysis. Data are shown as mean ± SEM (*n* = 7 per group) and analyzed by unpaired Student's *t* test with ***, *P < *0.001 in comparison with the CON group and **^###^**, *P < *0.001 in comparison with the SAP group.

Current studies suggest that an imbalance of intestinal microbiota may be involved in the pathogenesis of SAP-ALI and that it is closely related to the severity of the disease (13, 14, 33). Similar results were obtained in our study of disease phenotypes, as summarized in Fig. 1 (Abx+SAP group). To further characterize the alterations in gut microbiota during SAP-ALI and the effects of QYD on them, we performed amplicon sequencing analysis of the 16S rRNA gene using the cecal feces of mice (7 samples per group). Compared to the CON group, the number of operational taxonomic units (OTUs) in the gut microbiota, the homogeneity of species, and the relative abundance of intrinsically dominant bacteria in the gut were significantly lower after the Abx cocktail intervention, which is in line with the design aims of our study (see Fig. S1A-C in the supplemental material). The abundance and diversity of gut bacteria were significantly altered in the SAP group compared to wild-type mice, with an overall higher *Firmicutes*/*Bacteroidetes* ratio (Fig. 2A–C). All indexes, primarily total species, Shannon index, Chao1, and Simpson index, were all lower than those in the CON group (Fig. 2E–G, Fig. S1E). This suggests that bacterial diversity in the intestine is reduced during SAP-ALI. Mice in the Abx+SAP group showed a significant decrease in overall abundance and diversity of gut microbes after five consecutive days of pregavaged Abx intervention, which would lead to a decrease in the initial levels of postestablishment of the SAP model. Although the proportion of *Firmicutes*/*Bacteroidetes* was increased in the Abx+SAP group compared with the other groups, the attenuated organ damage and systemic inflammatory response may be due to a reduction of the relative concentration of displaced bacteria along the gut-lung axis (Fig. 2A–C). The composition of the gut microbiota in the QYD group was similar to that of the CON group, although the alpha diversity of bacterial species in the gut did not show a significant increase after QYD treatment and was verified via species richness-based principal-component analysis (PCA) as well as weighted Unifrac-based principal coordinates analysis (PCoA) (Fig. 2H, Fig. S1F). At the genus level, a decrease in the relative abundance of potentially pathogenic genera (Escherichia, *Enterococcus*, Enterobacter, *Peptostreptococcus*, *Helicobacter*) and an increase in the relative abundance of SCFAs-producing genera (*Bacteroides*, *Roseburia*, *Parabacteroides*, *Prevotella*, *Akkermansia*) were found in the QYD-treated group compared to the SAP group (Fig. 2D, Fig. S1D and G). Functional prediction analysis based on PICRUSt2 showed that treatment with QYD increased the biosynthesis of fatty acids, indicating the possibility of increased SCFAs-producing bacteria (Fig. 2I). Furthermore, the differential analysis of LDA effect size (LEfSe) showed that *Enterococcus* and *Bacteroides* were the representative bacterial genera present in the SAP modeling group and QYD treated group, respectively (Fig. 2J and K). Based on our intestinal bacterial composition results, we hypothesize that the mechanisms of action for QYD in alleviating SAP-ALI symptoms may be related to its regulatory potential of gut microbiota, specifically the upregulating synthesis of SCFAs and enhancement of the intestinal barrier.

### The intestinal barrier was damaged during SAP-ALI, while QYD treatment restored gut barrier function and reduced intestinal permeability.

**FIG 3:**
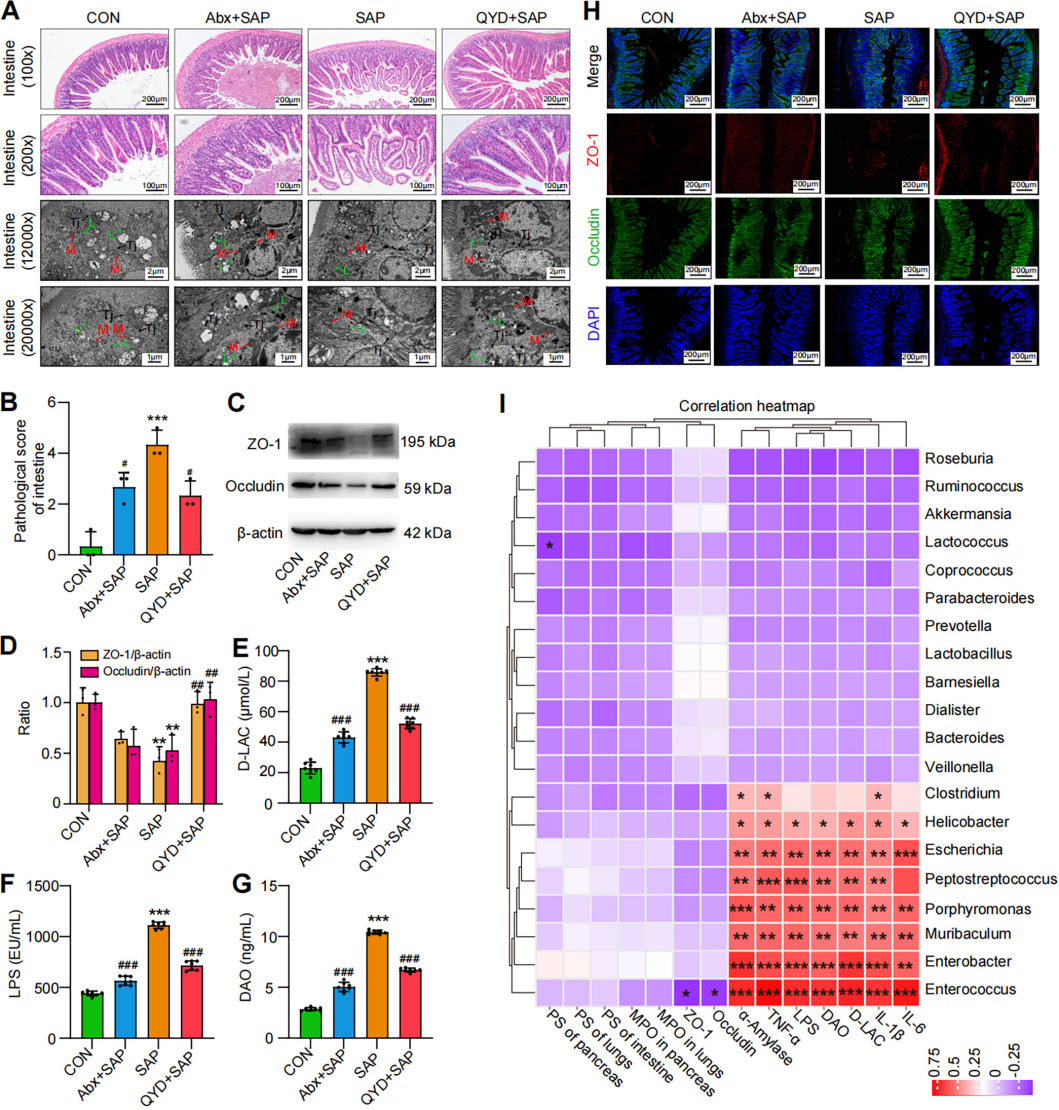
Abx intervention affected intestinal barrier function, while QYD treatment increased intestinal barrier protein expression and decreased intestinal permeability. (A) Representative H&E staining of intestine samples via microscope (100× and 200× magnification) and transmission electron microscopy (12,000× and ×20,000 magnification). L: lysosomal, M: mitochondria, Tj: tight junction. (B) The pathological score of the intestine (ileum). (C) Representative Western blotting images of ZO-1 and occludin. (D) Relative expression ratio of ZO-1/β-actin and occluding/β-actin. (E to G) Concentrations of d-LAC, LPS, and DAO in serum. (H) Immunofluorescence staining results for ZO-1 (red), occludin (green), and DAPI (blue), as well as merged images. Scale bar: 100 μm. (I) Heatmap of the correlations between intestinal bacteria (genus level) and pathological indices of SAP-ALI based on Spearman's correlation analysis. PS: pathological score. Data are representative images or shown as mean ± SEM for each group of mice (*n* = 7 per group) with at least three independent experiments and analyzed by unpaired Student's *t* test. *, *P < *0.05; **, *P < *0.01; ***, *P < *0.001 in comparison with the CON group and **^#^**, *P < *0.05; **^##^**, *P < *0.01; **^###^**, *P < *0.001 in comparison with the SAP group.

It has previously been reported that the gut is one of the most vulnerable organs during the progression of SAP and can amplify the inflammatory response throughout the body (34). SAP-ALI is usually accompanied by intestinal barrier dysfunction and slowed or even complete cessation of intestinal peristalsis (13). Therefore, we decided to investigate the functional changes of the intestinal barrier during SAP-ALI and the potential effects of QYD treatment on its pathological changes and permeability. Microscopic analysis of ileal tissue from SAP-ALI mice showed significant pathological damage compared to the CON group, revealing edema of the intestinal villi, subendothelial hemorrhage, infiltration of inflammatory cells, broken tight junctions between the cells, and lack of mitochondrial cristae (Fig. 3A and B). Furthermore, relative expression levels of tight junction proteins ZO-1 (Fig. 3H, red) and occludin (Fig. 3H, green) in the SAP mice model group were found to be decreased and irregular (Fig. 3C–D and H). Interestingly, mice in the Abx+SAP group exhibited decreased intestinal pathological damage scores and reduced levels of d-lactate (d-LAC), LPS, and diamine oxidase (DAO) compared to the SAP group (Fig. 3B and E–G). These data further illustrated that imbalances in the gut microbiota have the potential to influence the course of SAP-ALI. Compared to the SAP group, mice administered with QYD were found to have improved pathological damage to the intestine and expression levels of intestinal tight junction proteins (ZO-1 and occludin) (Fig. 3A–D, Fig. S2A and B). Similarly, decreased levels of d-LAC, LPS and DAO were found in the QYD-treated group, indicating relief of intestinal mucosal damage, reduced intestinal permeability, and the incidence of endotoxemia (Fig. 3E–G). Finally, we analyzed the correlation between significantly different genera and SAP-ALI disease phenotypes in mice (Fig. 3I). Our results showed that Escherichia, *Enterococcus*, Enterobacter, and *Peptostreptococcus* were positively correlated with the pancreatic damage (α-amylase), the systemic inflammatory responses (IL-1β, IL-6, and TNF-α), the intestinal permeability (DAO and d-LAC), and release of bacterial endotoxins (LPS), and negatively correlated with the expression of intestinal barrier proteins (ZO-1 and occludin). SCFAs-producing bacteria (*Bacteroides*, *Roseburia*, and *Prevotella*) presented positive correlations with intestinal barrier functions to some extent and negative correlations with systemic inflammation. Given these results, we hypothesized that the recovery of intestinal barrier function, pathological damage, and reduction of systemic inflammatory response in SAP-ALI after QYD treatment may be closely associated with SCFAs pathways.

### An altered metabolic profile for SCFAs was observed under SAP-ALI, propionate and butyrate may be potential key SCFAs regulated by QYD treatment.

**FIG 4:**
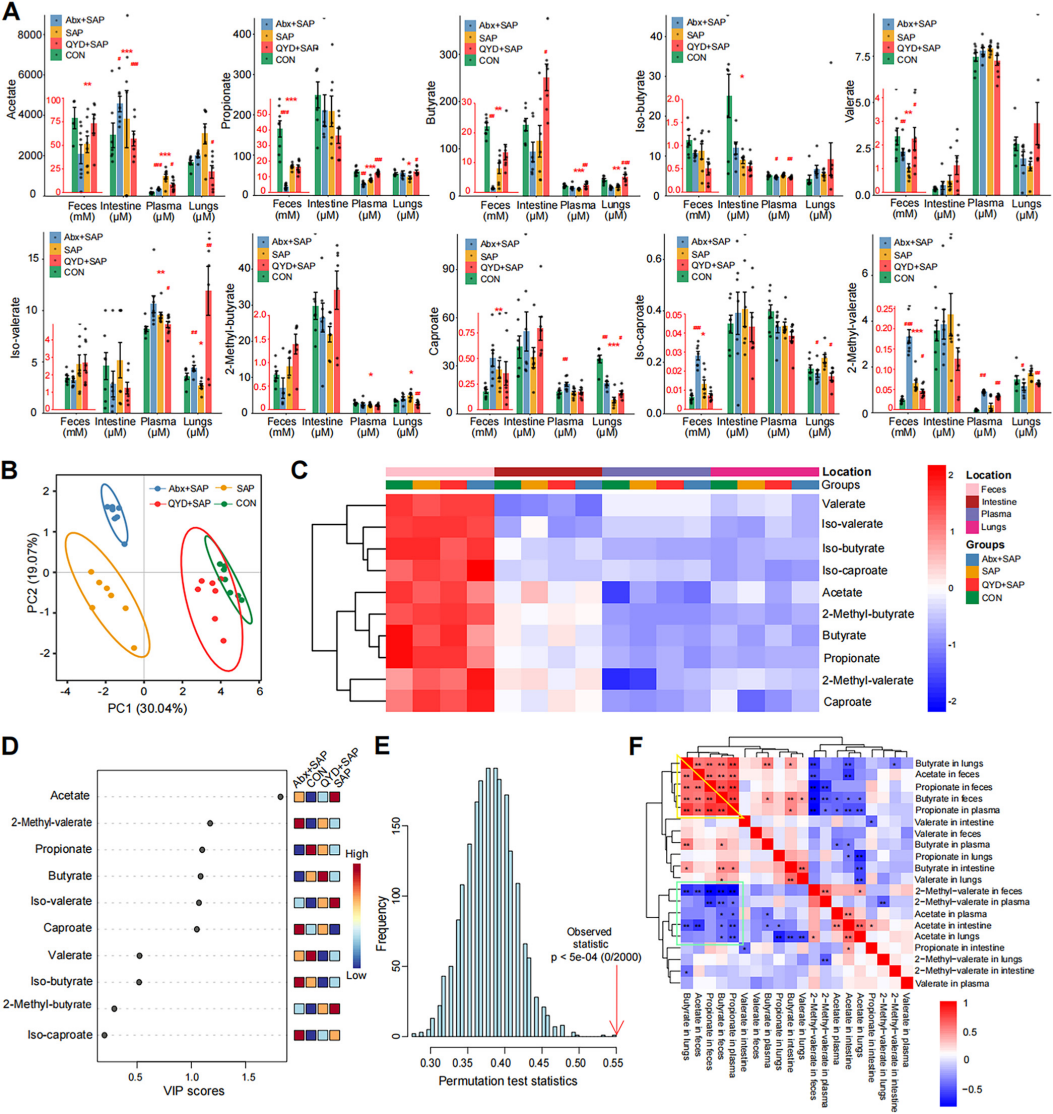
Differences in the concentration of SCFAs in sites related to the gut-lung axis among the groups. (A) The histogram of SCFAs concentrations in plasma, feces, lungs, and intestine. (B) PCA analysis of SCFAs was performed and shown in a circle plot according to a 95% confidence interval (CI). (C) Heatmap of SCFAs in different groups under different sample types. (D) The VIP score calculated by MetaboAnalyst (V5.0) with a score higher than 1 indicates that the SCFA is representative of the subgroup. (E) Construction of the PLS-DA model, and a *P < *0.05 indicates an ideal model fit. (F) Correlation heatmap of SCFAs under different sample types and groups was drawn according to the Spearman correlation analysis. Data are shown as mean ± SEM for each group of mice (*n* = 7 per group) and analyzed by unpaired Student's *t* test. *, *P < *0.05; **, *P < *0.01; ***, *P < *0.001 in comparison with the CON group and **^#^**, *P < *0.05; **^##^**, *P < *0.01; **^###^**, *P < *0.001 in comparison with the SAP group.

Remote regulation of SCFAs along the gut-lung axis plays an important role in the protective mechanism of SAP-ALI (14, 35). To compare the metabolism profiles of SCFAs in SAP-ALI mice and those treated with QYD, we measured 10 common SCFAs in sites associated with the gut-lung axis (feces, plasma, intestine, and lungs), as shown in Fig. 4A. Results showed that levels of several dominant SCFAs, such as acetate, propionate, butyrate, and valerate, significantly decreased in the SAP-ALI group, and the total number of SCFAs varied among all groups (Fig. 4A and B). Apart from feces, the highest levels of SCFAs were found in the intestine, followed by plasma and lungs, which is consistent with the trend that SCFAs exert remote epigenetic regulation along the gut-lung axis (Fig. 4C). Variable importance in projection (VIP) scores were calculated by constructing a perform partial least-squares-discriminant analysis (PLS-DA) model to assess the most representative SCFA in each group of mice (Fig. 4D). The permutation test results showed that the PLS-DA model was reliable and not overfitted (Fig. 4E). VIP scores showed that acetate, 2-methyl-valerate, propionate, butyrate, iso-valerate, and caproate could be used as a potential marker to distinguish the four groups (Fig. 4D). Next, we performed Spearman's correlation analysis for SCFAs in the different tissues (Fig. 4F). Results showed that propionate and butyrate in the feces performed a significant positive correlation with that *in vivo* (intestine, plasma, and lungs), and the levels of which were consistent with the progressive decreasing trend of SCFAs along the gut-lung axis. While acetate level in feces was negatively correlated with the level in other sites, we deduced this might be related to internal anaerobic glycolysis (36). Furthermore, the concentration of 2-methyl-valerate in the feces was negatively correlated with the acetate, propionate, and butyrate levels (Fig. 4F).

**FIG 5:**
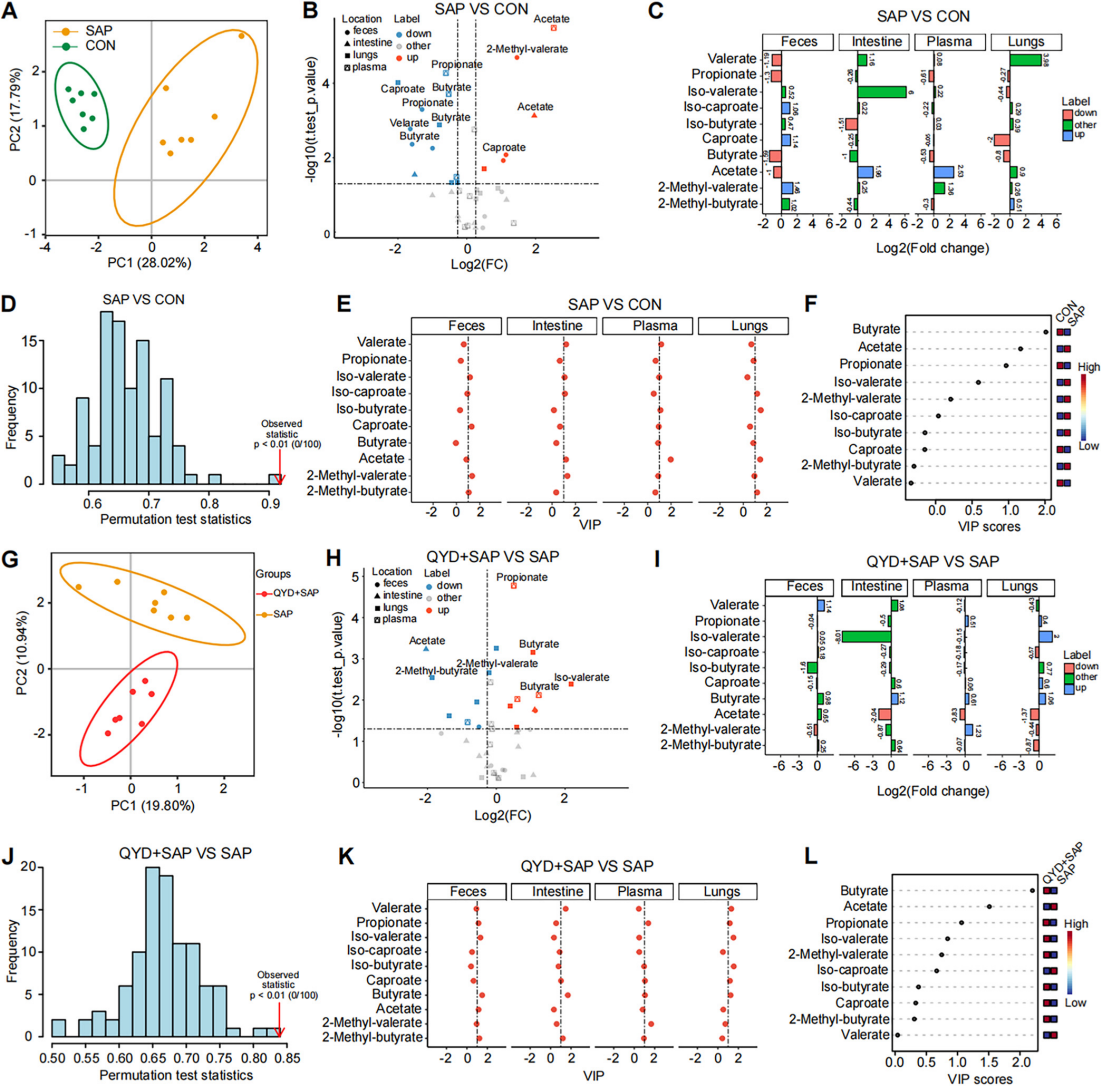
Pairwise comparison results of SCFAs in different groups. (A, G) PCA analysis of SCFAs in each group. (B, H) Volcano plot of SCFAs in each group. (C, I) Fold change plots of SCFAs in feces, plasma, intestine, and lungs of each group. (D, J) Permutation test charts of SCFAs using the PLS-DA model, and a *P < *0.05 indicates an ideal model fit. (E, K) The VIP score of SCFAs in feces, intestine, plasma, and lungs of each group. (F, L) The total VIP scores of SCFAs were calculated in each group.

To further investigate the detailed differences in the metabolism of SCFAs under SAP-ALI disease state and after QYD treatment, a pairwise comparison was performed to identify the most representative SCFAs in each group. The gut microbiota composition differs by subgroup (Fig. 5A and G). And we found a downregulation of propionate and butyrate in all four samples compared to the CON group under SAP-ALI (Fig. 5B to C). The results of the VIP score based on the PLS-DA model showed that acetate was the most representative SCFA, while the SCFAs with higher concentrations in the CON group were butyrate and propionate (Fig. 5D–F). Compared to the SAP group, propionate and butyrate were increased in plasma and lungs, and acetate was decreased in the QYD-treated group (Fig. 5H and I). Similarly, the results of the VIP score based on the PLS-DA model showed that acetate was the most representative in distinguishing the SAP group, while the typical SCFAs in the QYD treatment group were butyrate and propionate, especially butyrate (Fig. 5J–L). These results suggest a decrease in propionate, and butyrate is a major characteristic for mice suffering from SAP-ALI, which can be increased via QYD treatment.

### The mechanism of QYD in alleviating SAP-ALI may be involved in regulating the AMPK/NF-κB/NLRP3 pathway by SCFAs.

**FIG 6:**
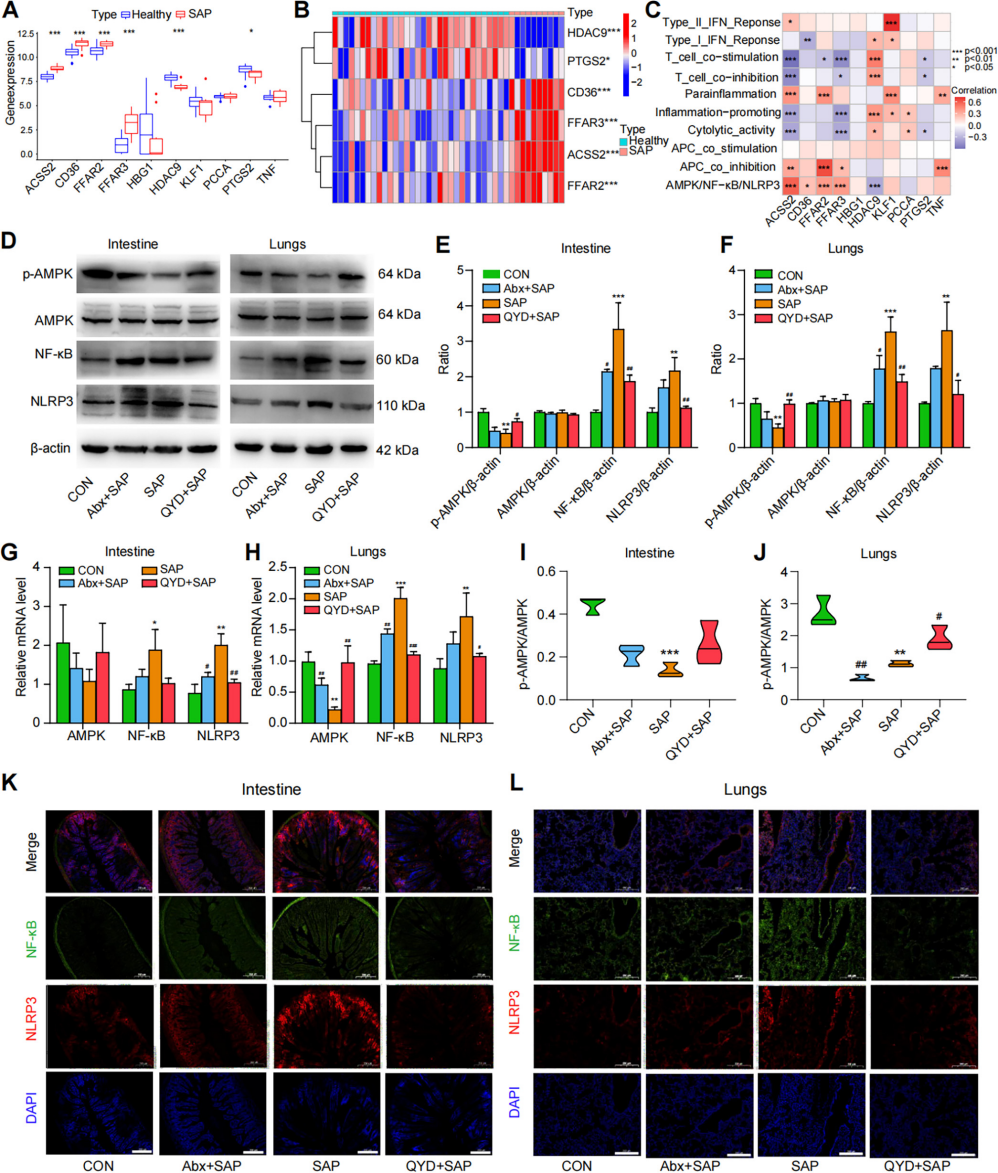
QYD may alleviate SAP-ALI through the SCFAs-mediated AMPK/NF-κB/NLRP3 pathway. (A) Boxplots of the differences in the relevant genes (GeneCards TOP 10) of SCFAs in SAP patients and control subjects based on the GEO database. (B) Heatmap of differences in genes related to SCFAs between SAP patients and controls based on GEO data set analysis. (C) Heatmap and Spearman’s correlation test of the enrichment scores for different pathways with SCFAs-related genes (GeneCards TOP 10) in SAP patients (by ssGSEA analysis) based on the GEO data set. (D) Representative Western blotting images of p-AMPK, AMPK, NF-κB, and NLRP3 in the intestine and lungs. (E and F) Relative expression ratios of p-AMPK, AMPK, NF-κB, and NLRP3 with β-actin in the intestine and lungs. (G and H) Relative AMPK, NF-κB, and NLRP3 mRNA levels in the intestine and lungs. (I and J) Relative expression ratio of p-AMPK to AMPK in the intestine and lungs. (K and L) Immunofluorescence staining results for NF-κB (green), NLRP3 (red), and DAPI (blue) in intestine and lungs, as well as their merged images. Scale bar: 100 μm. Data are representative images with at least three independent experiments and analyzed by unpaired Student's *t* test. *, *P < *0.05; **, *P < *0.01; ***, *P < *0.001 in comparison with the CON group and **^#^**, *P < *0.05; **^##^**, *P < *0.01; **^###^**, *P < *0.001 in comparison with the SAP group.

It has been reported that SCFAs have the potential to mediate the phosphorylation of the AMPK pathway via binding to G protein-coupled receptors on the cell surface, thus inhibiting the NF-κB/NLRP3 pathway (37, 38). Results from our previous studies have suggested that the activation of NLRP3 inflammasome plays a vital role in the pathogenesis of SAP-ALI (39). To further investigate the downstream mechanisms of QYD in the regulation of SCFAs, we first performed gene correlation analysis and single-sample gene enrichment analysis (ssGSEA) based on the GEO database (accession number GSE194331) to explore gene association related to the metabolism of SCFAs within the context of the AMPK/NF-κB/NLRP3 pathway in SAP patients. We discovered that genes involved in the epigenetic regulation of SCFAs, namely, *FFAR2*, *FFAR3*, *CD36*, *ACSS2*, and *HDAC9*, were significantly regulated in SAP patients (Fig. 6A and B). Using the ssGSEA and Spearman's rank correlation test of genes, enrichment fraction results showed that the downstream receptor genes of SCFAs perform a clear correlation with the AMPK/NF-κB/NLRP3 pathway, which indirectly demonstrates the possible microregulation of the AMPK/NF-κB/NLRP3 pathway by SCFAs during SAP-ALI (Fig. 6C).

We next compared the differences in protein and mRNA expression associated with the AMPK/NF-κB/NLRP3 signaling pathway in each group. Western blot (Wb) results showed that mice with SAP-ALI have downregulated the p-AMPK expression and upregulated NF-κB and NLRP3 expression in both intestine and lungs compared to the CON group (Fig. 6D–F). The levels of p-AMPK were increased, and the concentrations of NF-κB and NLRP3 were decreased in the intestine and lung tissue of the QYD-treated group compared with the SAP group (Fig. 6D–F). The quantitative reverse transcription-PCR (RT-qPCR) data showed the same results. We observed that QYD treatment increased the content of total AMPK mRNA and decreased the levels of NF-κB and NLRP3 mRNA in the intestine and lungs compared to the SAP group (Fig. 6G and H). The immunofluorescence staining results demonstrated that the fluorescence intensity of p-AMPK was diminished, and that of NLRP3 and NF-κB was enhanced in SAP-ALI mice (Fig. 6K and L, Fig. S3A to D). Compared with the SAP group, the QYD-treated group tended to revert to the CON group regarding immunofluorescence staining and an increased p-AMPK/AMPK ratio, especially in the lungs (Fig. 6I and J). Interestingly, the expression of p-AMPK was increased, and the levels of NF-κB and NLRP3 were decreased in the Abx+SAP group compared with the SAP-ALI group, which may be related to the reduced relative abundance of pathogenic bacteria following antibiotic gavage and warrants more in-depth study in the future. In conclusion, the results of these data suggest that activation of the AMPK/NF-κB/NLRP3 pathway is involved in the mechanism of SAP-ALI alleviation by QYD, which may be associated with increased synthesis of SCFAs.

### Correlations among intestinal bacteria, SCFAs, the AMPK/NF-κB/NLRP3 pathway, and SAP-ALI phenotypes.

**FIG 7:**
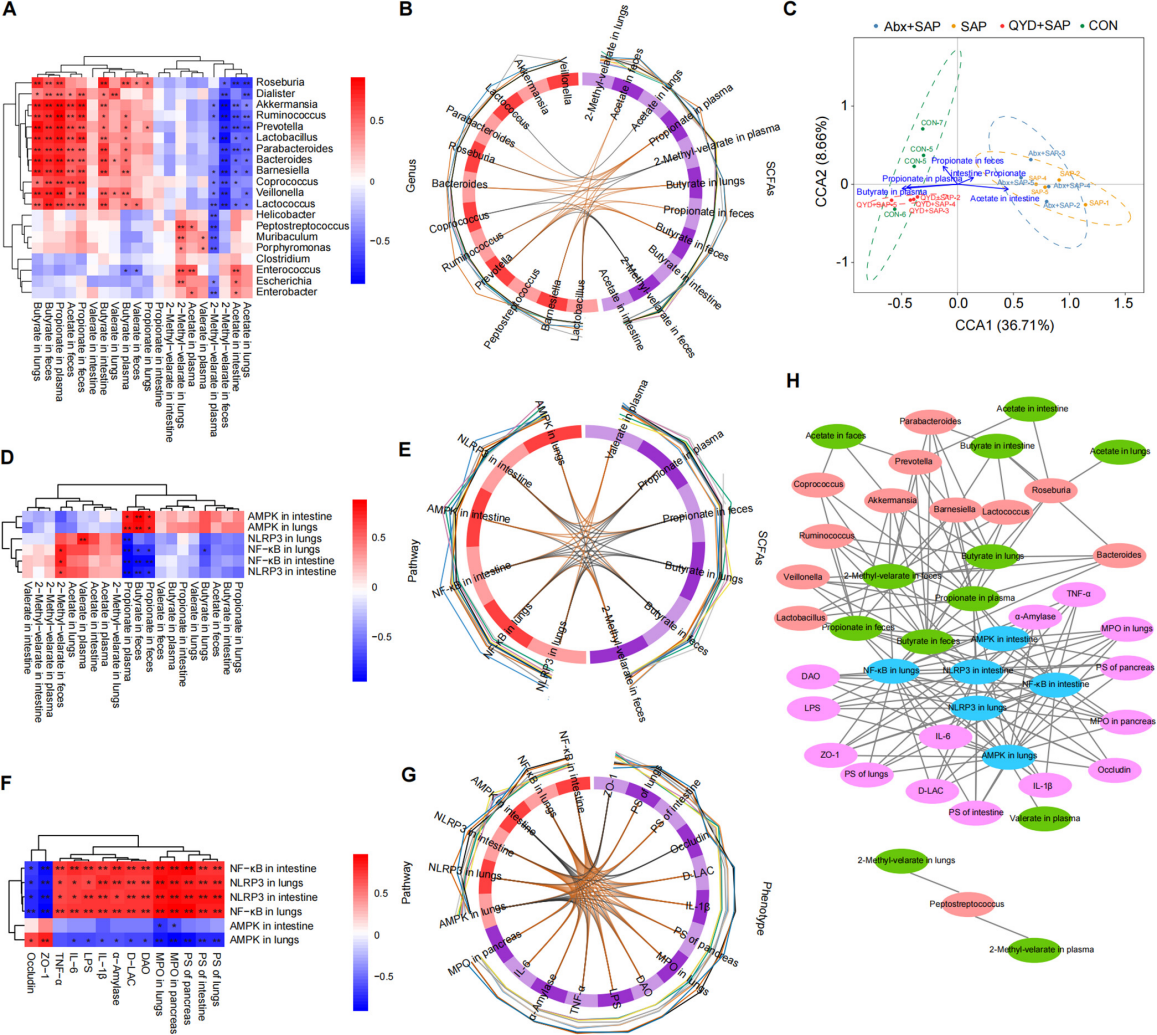
Correlation analysis between intestinal bacteria, SCFAs, AMPK/NF-κB/NLRP3 pathway, and SAP-ALI phenotype. (A) Heatmap of the correlation between intestinal bacteria and SCFAs based on Spearman correlation analysis. (B) Chord diagram of the correlations between the gut microbiome and SCFAs based on the block.splsda function analysis, the line in the circle represents the correlation coefficient between the bacteria and SCFAs greater or equal to 0.6. (C) CCA analysis. Arrows indicate SCFAs at different sites, dots indicate different samples, and the length of the arrow indicates the degree of correlation between SCFAs and the microbiota in that subgroup. (D) Heatmap of the correlations between SCFAs and the AMPK/NF-κB/NLRP3 pathway. (E) Chord diagram of the correlations between SCFAs and the AMPK/NF-κB/NLRP3 pathway. (F) Heatmap of the correlations between the AMPK/NF-κB/NLRP3 pathway and disease phenotypes of SAP-ALI. (G) Correlation chord diagram of the AMPK/NF-κB/NLRP3 pathway with disease phenotypes of SAP-ALI. (H) Network diagram showing intestinal bacteria, SCFAs, AMPK/NF-κB/NLRP3 pathways, and SAP-ALI disease phenotypes. Connected lines represent correlations greater than 0.6, with *P < *0.05. The asterisks in the heatmaps represent significance level. *, *P < *0.05; **, *P < *0.01.

Spearman's rank correlation analysis was used to determine the correlations between intestinal bacteria, SCFAs, the AMPK/NF-κB/NLRP3 pathway, and disease phenotypes of SAP-ALI mice. Correlation analysis of intestinal bacteria with SCFAs showed the relative abundance of *Roseburia*, *Akkermansia*, *Parabacteroides*, *Prevotella*, *Bacteroides*, and *Lactococcus* was positively correlated with the levels of butyrate and propionate, and negatively correlated with acetate (especially in lung tissue). However, Escherichia, *Enterococcus*, and Enterobacter were positively correlated with acetate and negatively correlated with 2-methyl-valerate in plasma (Fig. 7A). Chord diagrams showed that *Roseburia* was positively correlated with butyrate and negatively correlated with acetate in the lungs (Fig. 7B). The results of canonical correlation analysis (CCA) showed that butyrate and propionate in the plasma were the most closely correlated with the gut microbiota in the CON and QYD-treated groups (Fig. 7C). Next, we explored the correlation between SCFAs and AMPK/NF-κB/NLRP3 signaling pathway. Our results revealed that the expression of SCFAs (propionate and butyrate) in intestine and lung tissue was positively correlated with AMPK mRNA expression and negatively correlated with NF-κB and NLRP3 mRNA content, while acetate showed the exact opposite trend (Fig. 7D). Similar results were found in the chord diagrams, especially the correlation of butyrate with AMPK/NF-κB/NLRP3 pathways in lung tissue (Fig. 7E). In addition, correlation analysis of the pathways and phenotypes showed that AMPK mRNA levels were positively correlated with intestinal barrier proteins (ZO-1 and occludin) and negatively correlated with histopathological damage, inflammatory indicators, and intestinal permeability. In contrast, elevated NF-κB and NLRP3 mRNA levels implied a more severe systemic inflammatory response and pathological damage (Fig. 7F and G). With this in mind, we further explored the potential target genes of QYD based on network pharmacology. Our results showed that the QYD treatment could increase the synthesis of fatty acids and the biological activities of acetyl-CoA and fatty acyl-CoA, further supporting our hypothesis (see Fig. S4 in the supplemental material). Finally, correlation network plots were used to visualize the association between intestinal bacteria, SCFAs, AMPK/NF-κB/NLRP3 pathway, and SAP-ALI phenotypes posttreatment with QYD (Fig. 7H). The above results suggest that mechanisms of QYD in alleviating SAP-ALI may be involved in regulating gut microbiota and thus targeting short-chain fatty acids (propionate and butyrate)-mediated AMPK/NF-κB/NLRP3 pathway.

## DISCUSSION

Gut microbiota is considered an essential regulator of human health, and dominant anaerobic bacteria prevent direct contact of opportunistic pathogens with the intestinal epithelium by forming biofilms through colonization resistance (40, 41). Imbalanced gut bacteria in SAP are associated with intestinal barrier dysfunction and triggers a sustained proinflammatory response at sites associated with the gut-lung axis, which in turn leads to SAP-ALI (42–45). Therefore, regulation of intestinal microbiota may be an effective means of reducing the severity of SAP-ALI.

TCM is an ideal prospective culture medium for intestinal microecology containing curative effects, few side effects, and long-lasting efficacy (25). Due to low absorption, TCM creates suitable conditions for potential interactions between the gut microbiota and associated active components, which have the potential to exert effects on target organs (46). QYD is a TCM commonly used after surgery for the treatment of SAP. In previous studies, our team has shown the potential for QYD to treat SAP-ALI or SAP-related multiple organ dysfunction by exerting antiinflammatory and enhancing the intestinal barrier while simultaneously maintaining immune homeostasis (47, 48). Furthermore, QYD has been shown to mediate the recovery of intestinal dysbiosis in SAP, indicating its role in regulating intestinal bacteria (25).

Therefore, to investigate the role of gut microbiota in SAP-ALI and further explore the mechanisms of QYD, we established two mouse models: an SAP mouse model via injection of caerulein and LPS, as well as a pseudogerm-free mouse model via treatment with Abx cocktails. Results showed that combined Abx treatment yielded a lower pathology score in the pancreas and lung tissue compared to the SAP group, increased expression of tight junction proteins, reduced intestinal permeability, and decreased release of proinflammatory factors and endotoxins. This supports the role of gut microbiota in SAP-ALI pathology. Although the intestinal barrier was disrupted in the Abx+SAP group, the relative depletion of microbes lowered overall SAP-ALI severity, which may be related to the reduced level of the proinflammatory immune response (49, 50). After QYD treatment, lungs and pancreas pathology scores, overall intestinal permeability functions, and levels of proinflammatory factors and endotoxins were significantly lower, thus confirming a therapeutic effect in treating SAP-ALI symptoms. Bacterial abundance analysis showed that the relative abundance of pathogenic bacteria (Escherichia, *Lactococcus*, Enterobacter) decreased, while the relative abundance of SCFAs-producing bacteria (*Roseburia*, *Bacteroides*, *Parabacteroides*, *Akkermansia*) increased significantly posttreatment with QYD.

As a critical energy source for intestinal epithelial cells, SCFAs affect the proliferation, differentiation, renewal, and repair of cells as well as exert antiinflammatory and immunomodulatory functions in the intestine (23, 51). Other studies have shown that SCFAs have the potential to alleviate ALI in rats by reversing the abnormal expression of tight junction proteins and inhibiting the translocation of PAMPs, thereby reducing the production of proinflammatory cytokines and preventing immune cell infiltration (52). Our targeted metabolomic data on sites associated with the gut-lung axis revealed increased concentrations of multiple SCFAs, particularly propionate and butyrate, especially in feces, plasma, and lungs after QYD treatment. Furthermore, these data coincide with the trend of changes in intestinal bacteria.

AMPK is a crucial sensor for restoring cellular energy homeostasis, and it can regulate cellular metabolic and immune status during SAP through SCFA-mediated signaling (53, 54). Other studies have shown that AMPK can regulate autophagy in the intestines and lungs and inhibits oxidative stress and inflammatory responses (55–57). In addition, AMPK plays a vital role in regulating the biological functions of the mitochondria as well as the homeostasis of organelles (58). Phosphorylation and activation of AMPK significantly reduce oxidative damage to alveolar epithelial and endothelial cells via reduction of NF-κB expression and inhibition of NLRP3, which is closely related to the pathology of SAP-ALI (59, 60). Therefore, to identify potential downstream targets of SCFAs, we performed ssGSEA and Spearman's rank correlation analysis based on data from the GEO database. We discovered that SCFAs related genes (*FFAR2*, *FFAR3*, *ACSS2*) were correlated with the AMPK/NF-κB/NLRP3 signaling pathway. We then performed Western blot, qPCR, and immunofluorescence to verify these data. The results showed an increase in the phosphorylation of AMPK and a decrease in NF-κB and NLRP3 expression in the intestine and lungs. Correlation analysis between bacteria and SCFAs, SCFAs and AMPK/NF-κB/NLRP3 pathway, pathway, and disease phenotypes, were confirmed. These results suggest that remote regulation of the gut microbiota and SCFAs along the gut-lung axis plays a crucial role in the pathogenesis of SAP-ALI.

Although we made some progress in determining the mechanism of action for QYD protection from SAP-ALI, some limitations still need to be addressed for future investigations. First, our microbiome results were based on 16S rRNA sequencing data. The addition of a metagenomics-based high-throughput sequencing analysis could have revealed more information about the function of individual bacteria for further mechanistic studies. Furthermore, using fecal microbiota transplantation and germfree mice in future studies is more convincing to validate that QYD improves SAP-ALI by modulating the gut microbiota. However, our study does provide novel mechanistic data for QYD use as a potential strategy to treat SAP-ALI.

**FIG 8:**
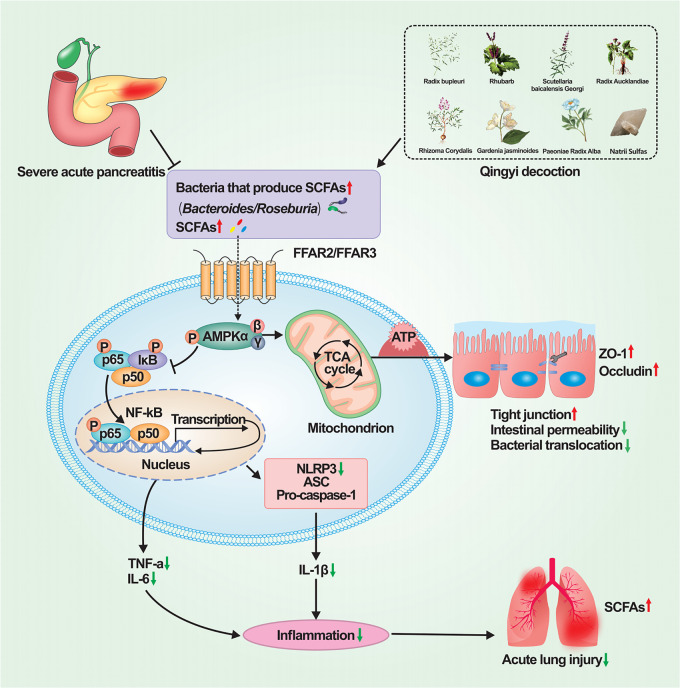
Mechanisms of Qingyi decoction in alleviating SAP-ALI via gut microbiota, which is probably associated with short-chain fatty acids-mediated AMPK/NF-κB/NLRP3 pathway. QYD treatment increased the relative abundance of SCFAs-producing bacteria, which would lead to a relative increase in the production of SCFAs. Combining SCFAs with G protein-coupled receptors in intestine and lungs cells can cause phosphorylation of AMPK, thereby inhibiting the expression of downstream target proteins NF-κB and NLRP3, and ultimately reducing histopathological damage and strengthening the intestinal barrier function, inhibiting the systemic sustained proinflammatory response to relieve SAP-ALI. AMPK, AMP (AMP)-activated protein kinase; ASC, apoptosis-associated speck-like protein; ATP, adenosine 5′-triphosphate; FFAR, free fatty acid receptor; IκB, inhibitors of kappaB; NLRP3, NOD-like receptor family pyrin domain containing 3; P, phosphorylation; p50, NF-kappa B1; p65, NF-kappa B RelA; SCFAs: short-chain fatty acids; TCA, tricarboxylic acid cycle; SAP-ALI, severe acute pancreatitis associated acute lung injury; ZO-1, zonula occludens-1.

In conclusion, we illustrated that QYD clearly alleviated SAP-ALI in a conventional mice model, supporting the therapeutic effect of this TCM. In parallel, mice treated with antibiotics alone did not develop significant SAP-ALI symptoms, suggesting the role of the gut microbiome in mediating SAP-ALI. The microbiome's role is further supported by the observation that both SCFAs and SCFAs-producing bacteria were enriched in QYD-treated conventional mice. Moreover, the increased SCFAs were associated with activating the AMPK/NF-κB/NLRP3 signaling pathway for host repair. A possible mechanism is shown in Fig. 8. Although preliminary and circumstantial, our results collectively lend support to a plausible mechanism for the therapeutic effect of QYD in SAP-ALI. That is, QYD could activate host repair by enriching SCFAs-producing bacteria for more SCFAs production. Further investigations are needed to test this hypothesis with more direct evidence.

## MATERIALS AND METHODS

### Materials and reagents.

The crude drugs (*Rhubarb* 20g, *Radix Bupleuri* 15g, *Radix Aucklandiae* 15g, *Paeoniae Radix Alba* 15g, *Natrii Sulfas* 10g, *Rhizome Corydalis* 15g, *Gardenia jasminoides* 15g and *Scutellaria baicalensis Georgi* 12g) for QYD were obtained from the First Affiliated Hospital of Dalian Medical University (Dalian, China). Neomycin sulfate and metronidazole were purchased from Solarbio Science and Technology Co. Ltd. (Beijing, China). Vancomycin hydrochloride and ampicillin sodium were obtained from Gypsy Biotechnology Co. Ltd. (Beijing, China). Caerulein and LPS were purchased from Sigma-Aldrich Co. (St. Louis, Mo). LC-MS-grade acetonitrile and formic acid were purchased from Thermosphere (St. Louis, USA). 3-nitrophenylhydrazine (3-NPH), and 1-ethyl-3-(3-dimethylaminopropyl) carbodiimide hydrochloride (EDC-HCl), as well as pyridine, were obtained from Mercklin (Shanghai, China). Distilled water was prepared using the Milli-Q System (Billerica, MA).

### Animals.

Specific pathogen-free (SPF) C57BL/6 mice (6 to 8 weeks, male) were purchased from the Laboratory Animal Center of Dalian Medical University (License number: SCXK [Liao] 2018-0003). All mice were housed in an SPF environment that included a 12-h day/night cycle at 20 to 22°C, 45% ± 5% humidity throughout the experiment period and allowed free access to food and water. Animal experiments were performed following experimental protocol AEE19003, approved by the Research and Animal Ethics Committee of Dalian Medical University (Dalian, China) and in compliance with national and international guidelines for the care and use of laboratory animals. All the mice were fed adaptively for 1 week before modeling.

### Establishment of the SAP-ALI mice model and groups.

Mice were randomly divided into four groups: including CON, severe acute pancreatitis (SAP), QYD-treated SAP (QYD+SAP), and cocktail-Abx-treated pseudogerm-free SAP (Abx+SAP) (*n* = 7 per group). An SAP-ALI model was established according to the previous protocol (49, 50). Briefly, the mice in SAP, QYD+SAP, and Abx+SAP groups were injected with caerulein (100 μg/kg) via intraperitoneal injection 10 consecutive times every hour, with one dose of LPS (10 mg/kg) administered with the last injection. The CON group was intraperitoneally injected with normal saline (NS) in the same volumes.

Mice in the Abx+SAP group were gavaged with a combination of four Abx (neomycin sulfate 200 mg/kg, metronidazole 200 mg/kg, ampicillin sodium 200 mg/kg, and vancomycin hydrochloride 100 mg/kg) for five consecutive days for the establishment of the SAP-ALI model (61). The mice in the QYD+SAP group were treated with oral administration of QYD (7.6 g crude drug per kg) immediately and 12 h postinjection with LPS. Animals were sacrificed via intraperitoneal injection of pentobarbital (100 mg/kg body weight) 24 h after postinjection of LPS. Blood, pancreas, distal ileum, lung tissue, cecal contents, and feces were collected for further analysis.

### Histopathological examination.

Pancreatic, ileal, and lung tissues were fixed overnight in 10% PBS-buffered formalin and embedded in paraffin wax. Sections were obtained as 5-μm thick blocks and stained with hematoxylin and eosin (H&E) solution. Images were captured via an optical microscope (Olympus, Japan) with a magnification of ×100 and ×200. Three fields of view were randomly selected for analysis for each tissue section, independently scored, and subsequently averaged. To evaluate the histopathological degree of edema, inflammation, vacuolation, and hemorrhagic necrosis, pathological indexes were scored as previously described, with scores ranging from 0 to 16 (total score for pancreas and lungs) representing the sum of scores for each group (41, 62–64). The pathology score of the intestine ranged from 0 to 5, as previously described (64, 65). Table S1 to 3 in the supplemental material contains the scoring criteria.

### Evaluation of enzyme activities by enzyme-linked immunosorbent assays.

Peripheral blood was collected via the fundus venous plexus puncture of mice. The supernatant (serum) was collected for enzyme-linked immunosorbent assay (ELISA) analysis after centrifugation at 3,000 rpm for 10 min at 4°C. Levels of α-amylase, IL-1β, IL-6, TNF-α, d-LAC, LPS, and diamine oxidase were analyzed according to the manufacturer's instructions. Mouse α-amylase, d-LAC, diamine oxidase, and endotoxin ELISA kits were purchased from Meibiao Biotechnology Co. Ltd. (Jiangsu, China). Mouse IL-1β, IL-6, and TNF-α ELISA kits were obtained from Elabscience Biotechnology Co. (Wuhan, China).

### Analysis of Wb.

First, 50 mg of ileal and lung tissues were homogenized via high-throughput tissue lysis (Jingxin, Shanghai, China). According to the manufacturer's instructions, total protein was extracted using an active protein extraction kit (KeyGEN BioTECH, Nanjing, China). After protein quantification via bicinchoninic acid, proteins were separated by sodium dodecyl sulfate-polyacrylamide gel electrophoresis (SDS-PAGE) and transferred to polyvinylidene difluoride (PVDF) membranes. Membranes were blocked in 5% skim milk for 2 h at room temperature by gentle shaking. Then, membranes were incubated with primary antibodies at a dilution ratio of 1:1,000 and 4°C overnight. The following antibodies were used: anti-ZO-1 (21773-1-AP) and anti-NF-κB (10745-1-AP) from (Proteintech, Chicago, United States); anti-occludin (ab216327) from (Abcam, Cambridge, United Kingdom); anti-p-AMPK (AF3423) from (Affinity Biosciences, Jiangsu, China); anti-NLRP3 (A5652), anti-AMPK (A1229), and anti-β-actin (AC038) from (Abclonal Biotech, Wuhan, China).

Membranes were washed three times with Tris-buffered saline Tween 20 (TBST) and incubated in secondary antibody with HRP-conjugated goat antirabbit IgG (H&L) (1:5,000, Abclonal Biotech) for 2 h. Membranes were exposed to enhanced chemiluminescence (ECL) and imaged with the Tanon 5200 imaging system (Shanghai, China). Image-Pro Plus 6.0 software was used to analyze the quantities of target proteins.

### RT-qPCR.

To quantify the transcriptional level of mRNA in the AMPK/NF-κB/NLRP3 pathway, RNAs in the gut and lung tissues were extracted using RNA extraction buffer (G3013, Servicebio, China), followed by reverse transcription using the Servicebio RT First Strand cDNA synthesis kit (G3330, Servicebio). cDNA was amplified using SYBR green qPCR master mix (Servicebio, G3320) buffer system via fluorescence quantitative PCR instrument (ABI 7500, USA). The results were normalized to the housekeeping gene *GAPDH* (ΔCt). Relative expression levels of the target genes were analyzed using the 2^−ΔΔCt^ method, with the relative level of target genes in the control group plotted as 1.0. The primer sequences are shown in Table 1.

**TABLE 1:** The sequences of primers

Genes	Forward primer (5′–3′)	Reverse primer (5′–3′)
GAPDH	CCTCGTCCCGTAGACAAAATG	TGAGGTCAATGAAGGGGTCGT
AMPK	CATTCCGAGATTTGGCTGTAGTTC	ATGTCTAGGTGGTTGTAGGTTTGC
NFKB	GAGAACGGCTGGCTGAAATG	CATTCCGAGATTTGGCTGTAGTTC
NLRP3	TAAGAACTGTCATAGGGTCAAAACG	GTCTGGAAGAACAGGCAACATG

### Immunofluorescence experiment.

To measure and visualize the expression of target proteins in tissues, including the distribution of myeloperoxidase (MPO) in lungs and intestine, the intestinal barrier proteins (ZO-1, occludin), and the critical proteins in AMPK/NF-κB/NLRP3 pathway in the intestine and lung tissues, we performed the immunofluorescence staining experiment using paraffin-embedded tissue slides. Briefly, the paraffin sections were deparaffinized, rehydrated, and sealed with bovine serum albumin to block the nonspecific antigens. Then, the diluted primary antibody was added and incubated overnight at 4°C in a humidified box. Primary antibodies against ZO-1 (21773-1-AP, Proteintech), NF-κB (10745-1-AP, Proteintech), occludin (ab216327, Abcam), NLRP3 (A5652, Abclonal), AMPK (A1229, Abclonal), p-AMPK (AF3423, Affinity), and antimyeloperoxidase antibody (MPO, GB12224, Servicebio) were used, respectively. After that, the slides were washed three times in PBS (pH 7.4) for 5 min each time, and then the secondary antibodies, namely, Cy3 conjugated Goat Anti-mouse IgG (H&L) (GB21301, Servicebio), FITC conjugated goat antimouse IgG (H&L) (GB22301, Servicebio) (1:300) were added and further incubated at room temperature for 50 min in the darkness. Finally, the cell nucleus was counterstained with DAPI, and the autofluorescence of the tissues was quenched. The images were observed and captured under a fluorescence microscope (NIKON ECLIPSE C1, Tokyo, Japan).

CaseViewer 2.4 (3DHISTECH, Budapest, Hungary) software was used for image processing, with three microscopic fields analyzed for each tissue section. Percent positive staining was calculated by Image J (National Institutes of Health).

### Preparation of samples for transmission electron microscope.

First, intestine and lung tissues were fixed with 1% osmic acid in 0.1 M phosphate buffer (PBS, pH 7.4) for 2 h at room temperature in the dark, and the samples were rinsed three times for 15 min each time. Tissues were dehydrated in graded ethanol (30%, 50%, 70%, 80%, 95%, and 100% for 20 min each) and then dehydrated twice in 100% acetone for 15 min each. Pure EMbed 812 resin (SPI, 90529-77-4) was poured into the embedding plate, and samples were placed into the embedding plate and maintained in a 37°C oven overnight. Then 60 to 80-nm thick ultrathin tissue sections were sliced, and the 150-mesh copper mesh was removed. The slices were placed in saturated alcohol solution with 2% uranium acetate for 8 min in a dark room for staining, then rinsed with 70% ethanol and ultrapure water three times. Next, 2.6% lead citrate was added and allowed to stain for 8 min. After washing and drying, the cuprum grids were placed on the grids board and dried overnight at room temperature. For analysis, images were obtained via HT7800 TEM (HITACHI Tokyo, Japan). Images were presented at 12,000× and 20,000× magnification.

### DNA extraction and 16S rRNA high-throughput sequencing.

Sequencing was performed with the help of LC-Bio Technologies (Hangzhou) Co., Ltd. According to the manufacturer's instructions, the total DNA from cecal fecal samples was extracted by E.Z.N.A. stool DNA kit (D4015, Omega Inc., Norcross, GA, USA). After quality control, total DNA was eluted in an elution buffer and stored for amplification. The V3-V4 region of bacterial 16S rRNA was amplified using primers 341F (5′-CCTACGGGNGGCWGCAG-3′) and 805R (5′-GACTACHVGGGTATCTAATCC-3′) (66). DNA products were confirmed via 2% agarose gel electrophoresis, then purified and quantified using the AMPure XT beads recovery kit (Beckman Coulter Genomics, Danvers, MA, USA) and Qubit dsDNA HS assay kit (Invitrogen, Carlsbad, CA, USA). The purified PCR product was evaluated using an Agilent 2100 Bioanalyzer (Agilent, Santa Clara, CA, USA) and library quantification kit (Kapa Biosciences, Woburn, MA, USA) before sequencing on the Illumina NovaSeq 6000 platform. The original raw data were spliced by overlapping quality control and chimera filtering to obtain 250-bp paired-end reads. A divisive amplicon denoising algorithm was used to dereplicate and establish OTUs, obtained through amplicon sequencing to conduct bacterial diversity analysis and annotation of species classification and differential analysis.

### Quantification of SCFAs by LC-MS/MS.

Quantification of SCFAs was conducted following the previous methods (67). SCFAs in feces, plasma, intestine, and lung tissues were pretreated by adding extraction reagent (acetonitrile) and centrifugation at 12,000 rpm for 5 min. The dried supernatant was dissolved in 40 μL acetonitrile-water solution (V:V = 1:1) and mixed internal standards. Then, 20 μL of 200 mM 3-nitrophenylhydrazine solution (in acetonitrile) and 10 μL of 120 mM 1-(3-Dimethylaminopropyl)-3-ethylcarbodiimide hydrochloride in acetonitrile-water solution (with 12% pyridine) were added for derivatization (40°C, 30 min). The reaction mixture was diluted to 800 μL, and the supernatant was prepared for analysis by LC-MS/MS (SCIEX, Framingham, MA). Chromatographic separation was performed via BEH C_18_ column (2.1 mm × 100 mm, 1.7 μm, waters, Billerica, USA), and the gradient elution conditions were: 0 to1.5 min, 25% acetonitrile; 1.5 to 6.0 min, 25%–45% acetonitrile; 6.0–8.5 min, 45%–70% acetonitrile; 8.5–9.5 min, 70%–98% acetonitrile; 9.5–11.5 min, 98% acetonitrile; 11.5–12.0 min, 98%–25% acetonitrile; 14 min, 25% acetonitrile. Mobile phase A consisted of 0.01% formic acid in water and mobile phase B consisted of acetonitrile with a flow rate 0.3 mL/min. The column temperature was kept at 40°C, and the injection volume was set at 5 μL.

Standards used were as follows: acetic acid (AA), propionic acid, butyric acid (BA), iso-butyric acid (IBA), 2-methyl-butyric acid (2-M-BA), valeric acid (VA), caproic acid (CA), and 2-methyl-velaric acid (2-M-VA) purchased from Mercklin (Shanghai, China); iso-valeric acid (IVA) and iso-caproic acid (ICA) were from Sigma-Aldrich (MO, USA). Stable isotope-labeled internal standards included D3-acetic acid; D7-butyric acid (D2-BA, 99.3% purity), D2-valeric acid (D2-VA, 99% purity) and D9-caproic acid (D2-CA, 99.2% purity), which were obtained from TRC (Toronto, Canada).

### Bioinformatics analysis.

Based on the OTUs clustering enrichment table, QIIME2 software (version 2021.2) was used to analyze alpha diversity and beta diversity values among the four groups. SILVA (release 138, https://www.arb-silva.de/documentation/release138/) and NT-16S databases were used for species annotation, and statistics were performed based on the OTUs abundance table for each sample. The confidence threshold for annotation was 0.7. To compare differences in the composition and functions of gut microbes in each group, R ggplot2 package was used for the analysis of column charts, stacked charts, circle charts, bubble charts, PCoA 2D diagrams, and branch evolution charts to visualize and compare the data of bacteria versus SCFAs. MetaboAnalyst5.0 was used to perform partial least-squares discriminant analysis (PLSDA) and perform variable importance in projection (VIP) score analyses to determine the contribution of SCFAs in each of the four groups. Correlation heatmapping (R pheatmap package), a chord diagram (R mixOmics package), canonical correlation analysis (CCA, R vegan package), a correlation circle plot (R mixOmics package), and a network plot (Cytoscape) were used to display the potential relationships between intestinal bacteria and metabolites, metabolites and pathways, and pathways and SAP-ALI phenotypes. Functions of the colonized bacteria were predicted based on marker gene sequences analyzed via Phylogenetic Investigation of Communities by Reconstruction of Unobserved States software (The Huttenhower Lab, MA, USA).

### ssGSEA and correlation tests.

The GSE194331 data set (including 32 healthy normal people and 10 SAP patients) was downloaded from the public gene expression omnibus (GEO) database (http://www.ncbi.nlm.nih.gov/geo). Based on the gene expression matrix of peripheral blood mononuclear cells of SAP patients, the gene set variation analysis (GSVA) package was used to perform ssGSEA analysis and calculate the enrichment scores for the AMPK/NF-κB/NLRP3 pathway. Subsequently, the Limma package (R language) was used to extract the expression level of SCFA metabolism-related genes (top 10 genes in GeneCards), and the correlations among these genes were calculated based on the Spearman rank correlation test.

### Statistical analysis.

The data are presented as the mean ± standard error of the mean (SEM), or median with 25th to 75th percentile range with at least 3 independent experiments. Statistical analyses were performed using R language (version 4.1.1; R Core Team, 2021). The data for the distribution of normality was tested using the Shapiro-Wilk test or Kolmogorov-Smirnov test. Differences between the four groups were tested using an independent Student's *t* test for two groups (with normally distributed variables) or one-way analysis of variance (ANOVA) for three or more groups (with normally distributed variables). Besides, Tukey’s test was used as *post hoc* after ANOVA. Spearman’s correlation was applied to the associations between intestinal bacteria and metabolites, metabolites and pathways, or pathways and phenotypes of SAP-ALI. The significance level was set to α = 0.05.

### Data availability.

The 16S rRNA sequencing data sets have been uploaded to the Sequence Read Archive (SRA) database under BioProject number PRJNA955760.
