# Supplementary material for: Mechanisms of Qingyi Decoction in Severe Acute Pancreatitis-Associated Acute Lung Injury via Gut Microbiota: Targeting the Short-Chain Fatty Acids-Mediated AMPK/NF-κB/NLRP3 Pathway
Source: Microbiol Spectr. 2023 Jun 20;11(4):e03664-22. doi: 10.1128/spectrum.03664-22 (PMC10434154; doi:10.1128/spectrum.03664-22)
Supplement: Supplemental file 1 — Supplemental methods, Tables S1 to S3, and Fig. S1 to S4. Download spectrum.03664-22-s0001.pdf, PDF file, 1.7 MB [file spectrum.03664-22-s0001.pdf]

SUPPLEMENTARY MATERIALS

Mechanisms of Qingyi decoction in Severe Acute Pancreatitis-Associated Acute Lung Injury via Gut Microbiota: Targeting Short-Chain Fatty Acids mediated AMPK/NF-κB/NLRP3 pathway

Zhengjian Wang,[a,b,c]# Jin Liu,[a,b,c]# Fan Li,[a,b,c] Shurong Ma,[a,c] Liang Zhao,[a,b] Peng Ge,[a,b,c] Haiyun Wen,[a,b,c] Yibo Zhang,[a,b,c] Xiaojun Liu,[b,c,d] Yalan Luo,[a,b,c] Jiaqi Yao,[b,c,d] Guixin Zhang,[a,b,c]* Hailong Chen,[a,c]*

SUPPLEMENTARY METHODS

Firstly, the ingredients of the QYD monomer were obtained (Rhubarb, Paeoniae Radix Alba, Radix Bupleuri, Scutellaria baicalensis Georgi, Radix Aucklandiae, Gardenia jasminoides) in Traditional Chinese Medicine Systems Pharmacy Database and Analysis Platform (TCMSP) (http://LSP.nwu.edu.cn/tcmsp.php), and the active ingredients were filtered according to the two indicators, namely oral bioavailability (greater than 30%) and drug-likeness (greater than 0.18) [1, 2]. SCFAs-related genes were obtained from the Genecards (www.genecards.org/), and then the dataset GSE194331 (including the transcriptome sequencing data of peripheral blood PBMCs of 32 control subjects and 10 SAP patients) was downloaded from GEO. Then, a total of 8154 differential genes were screened (according to logFC > 0.1 and adj.P .Val < 0.05), and these genes were regarded as SAP-related genes [3]. The top 20 up-regulated and down-regulated genes of the differential genes were included in the heatmap using the R pheatmap package to visualize the expression levels of these genes. In the meantime, the R ggplot, ggrepel, and dplyr packages were used to obtain a volcano map. Perl (ActivePerl, version 5.24) was used to convert the active components of monomers into related target genes based on the UniProt database (http://www.uniprot.org/). A Venn diagram was drawn by R package, and 14 common genes were obtained by intersecting the SAP-related genes, SCFAs gene, and the target genes of SCFAs corresponding to each monomer. Next, Cytoscape v3.9.0 (www.cytoscape.org/) was used to draw a network regulation map of SAP-QYD-SCFAs-related genes [4]. Finally, a gene function enrichment analysis was

performed based on Gene Ontology (GO) and the Kyoto Encyclopedia of Genes and Genomes (KEGG), and then the R ggplot2 package was used for bubble charts for visualization.

Table S1. The pathological score criteria of pancreas.

Condition	Score	Description
Edema	0	Absent
	1	Diffuse expansion of interlobar septae
	2	Same as 1 + diffuse expansion of interlobular septae
	3	Same as 2 + diffuse expansion of interacinar septae
	4	Same as 3 + diffuse expansion of intercellular spaces
Inflammation and perivascular infiltrate	0	0-1 intralobular or perivascular leukocytes/ HPF
	1	6-10 intralobular or perivascular leukocytes/ HPF
	2	16-20 intralobular or perivascular leukocytes/ HPF
	3	26-30 intralobular or perivascular leukocytes/ HPF
	4	>35 leukocytes/HPF or confluent microabscesses
Hemorrhage and fat necrotis	0	Absent
	1	2 foci
	2	4 foci
	3	6 foci
	4	8 foci
Acinar necrosis	0	Absent
	1	Diffuse occurrence of 1-4 necrotic cells/HPF
	2	Diffuse occurrence of 5-10 necrotic cells/HPF
	3	Diffuse occurrence of 11-16 necrotic cells/HPF (Foci ofconfluent necrosis)
	4	> 16 necrotic cells/HPF (Extensive confluent necrosis)

Table S2. The pathological score criteria of intestine.

Grade	Pathologic score
0	Normal mucosal villi
1	Development of subepithelial Gruenhagen’s space at the apex of the villus
2	Extension of the subepithelial space with moderate lifting of the epithelial layer from the lamina propria
3	Massive epithelial lifting down the sides of villi, possibly with a few denuded tips
4	Denuded villi with the lamina propria and dilated capillaries exposed
5	Digestion and disintegration of the lamina propria, hemorrhage, and ulceration

Table S3. The pathological score criteria of lungs.

Condition	Score	Description
**Neutrophil Infiltration**	0	No or very mild
	1	Mild
	2	Moderate
	3	Severe
	4	Very severe
**Edema**	0	No or very mild
	1	Mild
	2	Moderate
	3	Severe
	4	Very severe
**Disorganization Of Lung Parenchyma**	0	No or very mild
	1	Mild
	2	Moderate
	3	Severe
	4	Very Severe
**Hemorrhage**	0	No or very mild
	1	Mild
	2	Moderate
	3	Severe
	4	Very severe

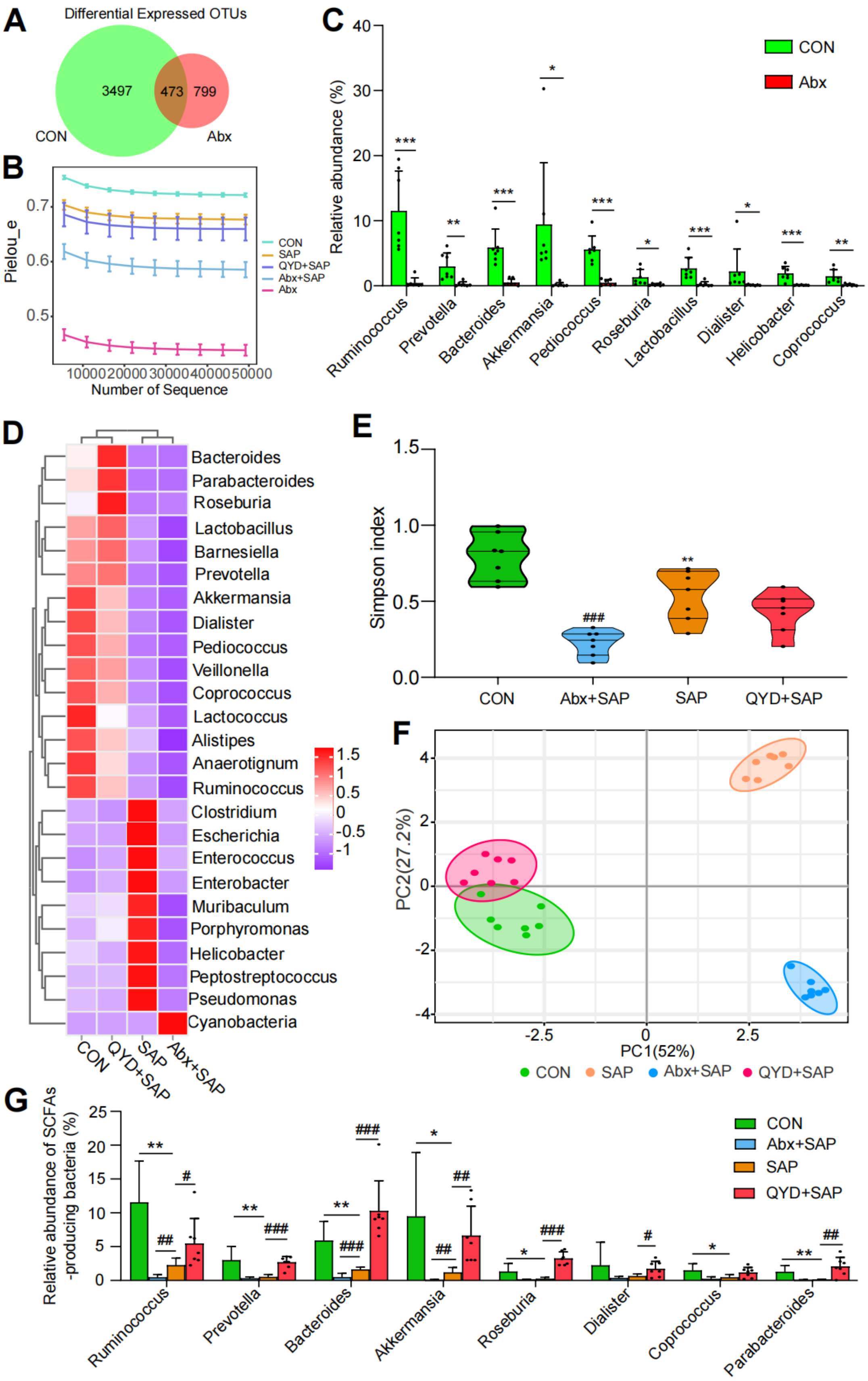
A
Differential Expressed OTUs
3497
473
799
CON
Abx
B
Pielou_e
Number of Sequence
CON
SAP
QYD+SAP
Abx+SAP
Abx
C
Relative abundance (%)
CON
Abx
Ruminococcus
Prevotella
Bacteroides
Akkermansia
Pediococcus
Roseburia
Lactobacillus
Dialister
Helicobacter
Coprococcus
D
Bacteroides
Parabacteroides
Roseburia
Lactobacillus
Barnesiella
Prevotella
Akkermansia
Dialister
Pediococcus
Veillonella
Coprococcus
Lactococcus
Alistipes
Anaerotignum
Ruminococcus
Clostridium
Escherichia
Enterococcus
Enterobacter
Muribaculum
Porphyromonas
Helicobacter
Peptostreptococcus
Pseudomonas
Cyanobacteria
CON
QYD+SAP
SAP
Abx+SAP
E
Simpson index
CON
Abx+SAP
SAP
QYD+SAP
F
PC2(27.2%)
PC1(52%)
CON
SAP
Abx+SAP
QYD+SAP
G
Relative abundance of SCFAs -producing bacteria (%)
CON
Abx+SAP
SAP
QYD+SAP
Ruminococcus
Prevotella
Bacteroides
Akkermansia
Roseburia
Dialister
Coprococcus
Parabacteroides

153

Figure S1. The composition and diversity analysis results of gut microbiota based on 16S rDNA sequencing. (A) Venn diagram of the CON and Abx groups based on OTU abundance. The numbers represent the values of OTUs that can be detected in all mice in a group. (B) Sparsity curves based on the pielou-e index showing species richness of each group. (C) The histogram shows the difference in the relative abundance of the TOP 10 genera between the CON and Abx groups. (D) Clustering heatmaps of bacteria at genus level in different groups. (E) Alpha diversity analysis of intestinal bacteria at the genus level (Simpson index). (F) PCA plot based on weighted unifrac distance matrix analysis of top25 bacteria. (G) The histogram shows the differences in the major SCFAs-producing genera in each group. Data are shown as mean ± SEM (n=7 per group) and analyzed by unpaired student's t-test with *, $P < 0.05$; **, $P < 0.01$; ***, $P < 0.001$ in comparison with the CON group and $^{\#}$, $P < 0.05$; $^{\#\#}$, $P < 0.01$; $^{\#\#\#}$, $P < 0.001$ in comparison with the SAP group.

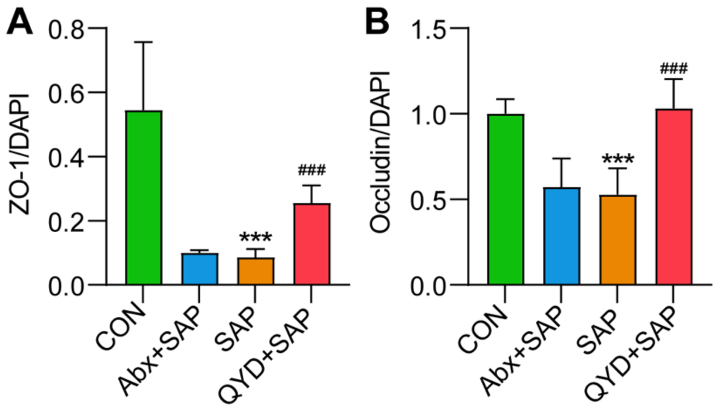

Figure S2. Semiquantitative results of tight junction protein in intestine. (A) Relative expression ratio of ZO-1/DAPI. (B) Relative expression ratio of occludin/DAPI. Data are shown as mean ± SEM (n=7 per group) and analyzed by unpaired student's t-test with ***, $P < 0.001$ in comparison with the CON group and ###, $P < 0.001$ in comparison with the SAP group.

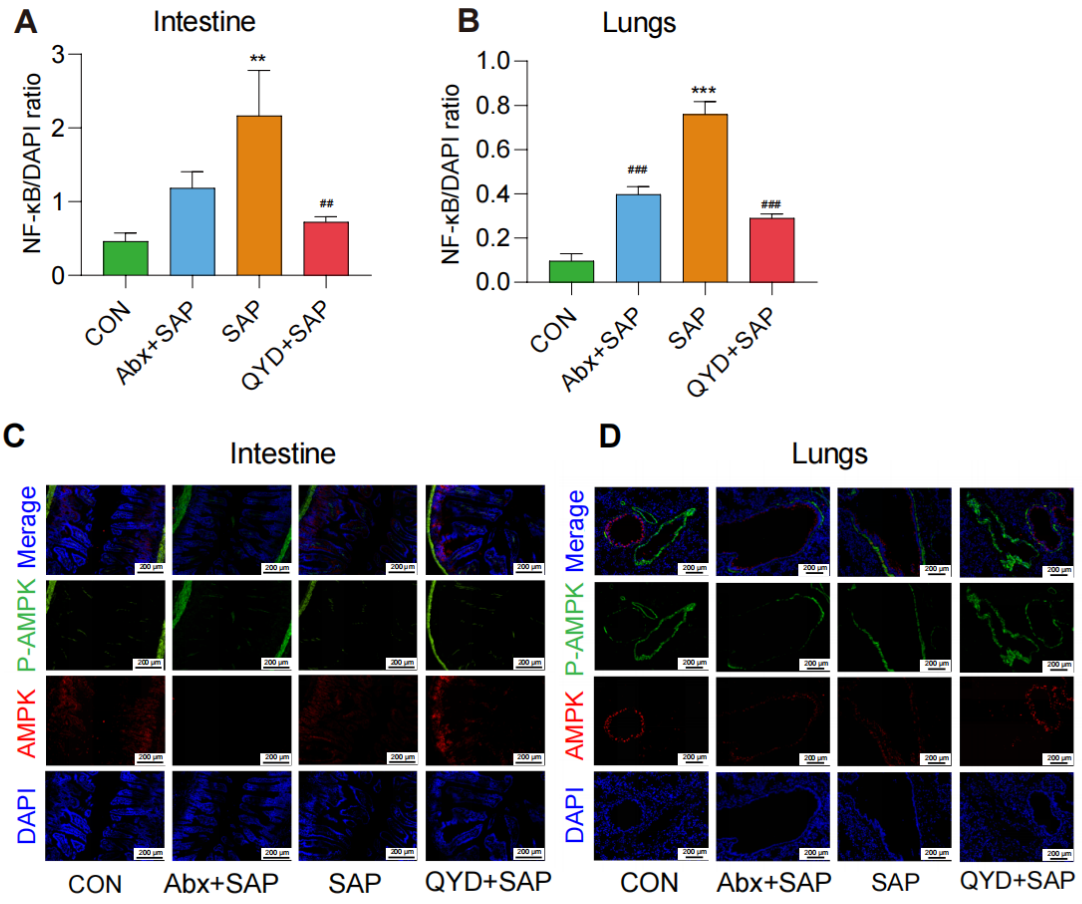

Figure S3. Semiquantitative results of NF-κB and immunofluorescence staining of AMPK-related proteins. (A) Relative expression ratio of NF-κB/DAPI in the intestine. (B) Relative expression ratio of NF-κB/DAPI in lungs. (C-D) Immunofluorescence staining results of AMPK (in red), p-AMPK (in green), DAPI (in blue), and the merge images, scale bar: 100 μm in lungs, and 200 μm in the intestine. Data are representative images with at least three independent experiments and analyzed by unpaired student's t-test. **, $P < 0.01$; ***, $P < 0.001$ in comparison with the CON group and $^{\#\#}$, $P < 0.01$; $^{\#\#\#}$, $P < 0.001$ in comparison with the SAP group.

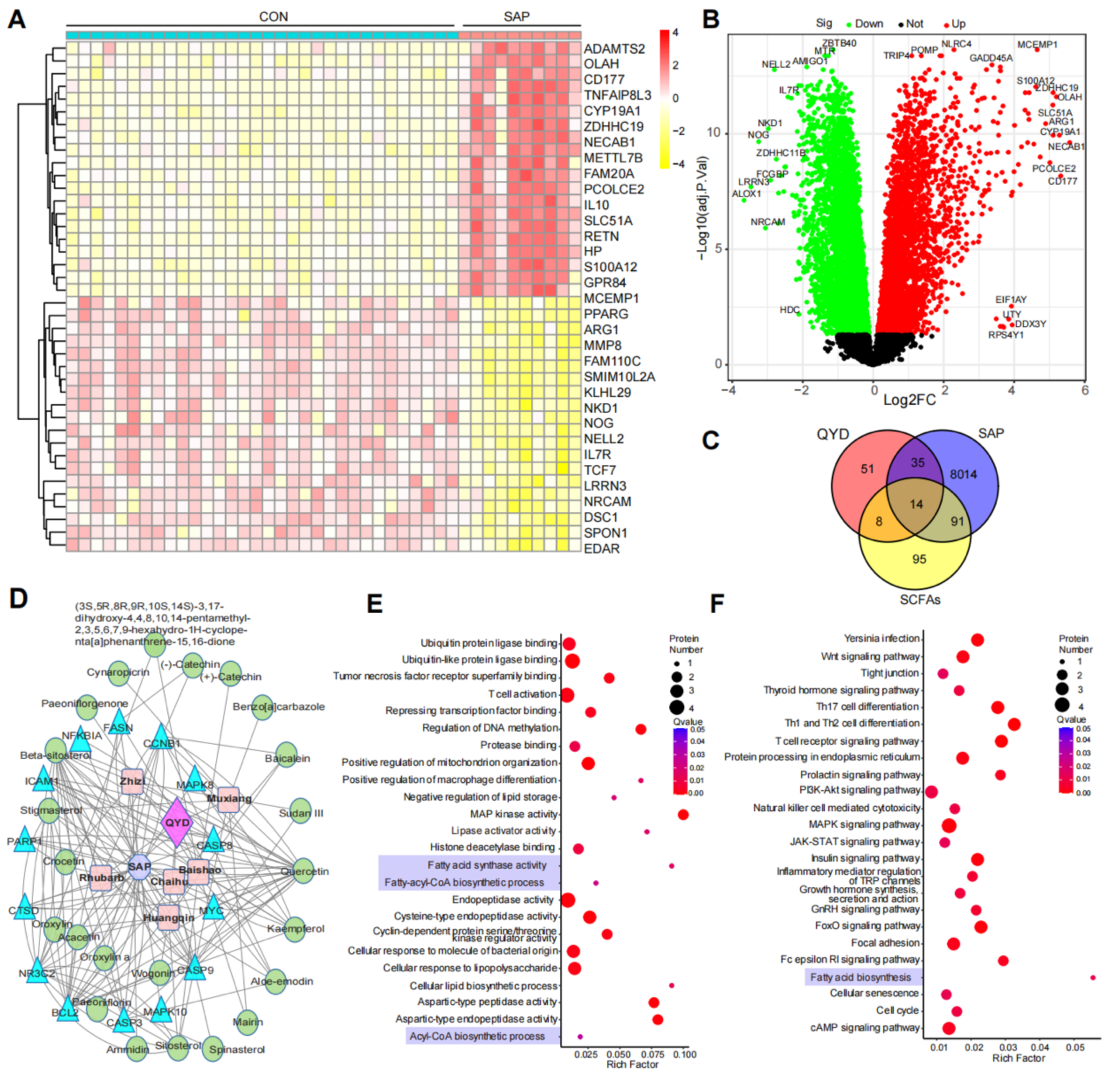

Figure S4. Relationship between QYD treatment and the major components of SCFAs in SAP patients based on network pharmacology. (A) Clustering heatmap of the up-regulated and down-regulated TOP20 genes in SAP patients and control subjects based on the GSE194331 dataset. (B) The volcano plot shows the differential genes between SAP patients and control subjects based on the GSE194331 dataset, according to the absolute value of log_2FC. The log_2FC value above 0.1 and P value less than 0.05 was set as the limit, and the most significant differential genes were marked with symbolic names. (C) The Venn diagram of the intersection of target genes in response to active ingredients of QYD, SAP-related genes, and SCFAs-related genes (acquired by

Genecards), obtaining a total of 14 intersecting genes. (D) Network map showing the SAP-related genes, target genes in response to QYD active ingredients, and SCFAs-related genes (acquired by Genecards). (E) Visualized bubble plot for functional enrichment analysis of intersecting genes based on the Gene Ontology (GO) database. (F) Bubble plot for functional enrichment analysis of intersecting genes based on the Kyoto Encyclopedia of Genes and Genomes (KEGG) database. Latin name: Zhizi, Gardenia jasminoides；Muxiang, Radix Aucklandiae; Chaihu, Radix Bupleur; Baishao, Paeoniae Radix Alba; Huangqin, Scutellaria baicalensis Georgi.

REFERENCES

1. Ru J, Li P, Wang J, Zhou W, Li B, Huang C, et al. TCMSP: a database of systems pharmacology for drug discovery from herbal medicines. J Cheminform (2014) 6:13. doi: 10.1186/1758-2946-6-13.
2. Zhang W, Chen Y, Jiang H, Yang J, Wang Q, Du Y, et al. Integrated strategy for accurately screening biomarkers based on metabolomics coupled with network pharmacology. Talanta (2020) 211:120710. doi: 10.1016/j.talanta.2020.120710.
3. Chen S, Sun Z, Deng W, Li G, Liu X, Zhang Z. Whole transcriptome analysis of long noncoding RNA in beryllium sulfate-treated 16HBE cells. Toxicol Appl Pharmacol (2022) 449:116097. doi: 10.1016/j.taap.2022.116097.
4. Piñero J, Saüch J, Sanz F, Furlong LI. The DisGeNET cytoscape app: Exploring and visualizing disease genomics data. Comput Struct Biotechnol J (2021) 19:2960-7. doi: 10.1016/j.csbj.2021.05.015.
